# Novel *Chloroflexi* genomes from the deepest ocean reveal metabolic strategies for the adaptation to deep-sea habitats

**DOI:** 10.1186/s40168-022-01263-6

**Published:** 2022-05-10

**Authors:** Rulong Liu, Xing Wei, Weizhi Song, Li Wang, Junwei Cao, Jiaxin Wu, Torsten Thomas, Tao Jin, Zixuan Wang, Wenxia Wei, Yuli Wei, Haofeng Zhai, Cheng Yao, Ziyi Shen, Jiangtao Du, Jiasong Fang

**Affiliations:** 1 Shanghai Engineering Research Center of Hadal Science and Technology, College of Marine Sciences, Shanghai Ocean University, Shanghai, China; 2 National Engineering Research Center for Oceanic Fisheries, Shanghai Ocean University, Shanghai, China; 3 Centre for Marine Science & Innovation and School of Biological Earth and Environmental Science, University of New South Wales, Kensington, Australia; 4 BGI-Shenzhen, Shenzhen, Guangdong China; 5 Tidal Flat Research Center of Jiangsu Province, Nanjing, Jiangsu China; 6 Laboratory for Marine Biology and Biotechnology, Qingdao National Laboratory for Marine Science and Technology, Qingdao, China; 7 Department of Natural Sciences, Hawaii Pacific University, Honolulu, HI USA

**Keywords:** *Chloroflexi*, Metagenome-assembled-genomes, Metabolic potential, Persistent organic pollutant, PAH, PCB, Dehalogenation, Feast-or-famine, Deep ocean, Hadal trenches

## Abstract

**Background:**

The deep sea harbors the majority of the microbial biomass in the ocean and is a key site for organic matter (OM) remineralization and storage in the biosphere. Microbial metabolism in the deep ocean is greatly controlled by the generally depleted but periodically fluctuating supply of OM. Currently, little is known about metabolic potentials of dominant deep-sea microbes to cope with the variable OM inputs, especially for those living in the hadal trenches—the deepest part of the ocean.

**Results:**

In this study, we report the first extensive examination of the metabolic potentials of hadal sediment *Chloroflexi*, a dominant phylum in hadal trenches and the global deep ocean. In total, 62 metagenome-assembled-genomes (MAGs) were reconstructed from nine metagenomic datasets derived from sediments of the Mariana Trench. These MAGs represent six novel species, four novel genera, one novel family, and one novel order within the classes *Anaerolineae* and *Dehalococcoidia*. Fragment recruitment showed that these MAGs are globally distributed in deep-sea waters and surface sediments, and transcriptomic analysis indicated their in situ activities. Metabolic reconstruction showed that hadal *Chloroflexi* mainly had a heterotrophic lifestyle, with the potential to degrade a wide range of organic carbon, sulfur, and halogenated compounds. Our results revealed for the first time that hadal *Chloroflexi* harbor pathways for the complete hydrolytic or oxidative degradation of various recalcitrant OM, including aromatic compounds (e.g., benzoate), polyaromatic hydrocarbons (e.g., fluorene), polychlorobiphenyl (e.g., 4-chlorobiphenyl), and organochlorine compounds (e.g., chloroalkanes, chlorocyclohexane). Moreover, these organisms showed the potential to synthesize energy storage compounds (e.g., trehalose) and had regulatory modules to respond to changes in nutrient conditions. These metabolic traits suggest that *Chloroflexi* may follow a “feast-or-famine” metabolic strategy, i.e., preferentially consume labile OM and store the energy intracellularly under OM-rich conditions, and utilize the stored energy or degrade recalcitrant OM for survival under OM-limited condition.

**Conclusion:**

This study expands the current knowledge on metabolic strategies in deep-ocean *Chlorolfexi* and highlights their significance in deep-sea carbon, sulfur, and halogen cycles. The metabolic plasticity likely provides *Chloroflexi* with advantages for survival under variable and heterogenic OM inputs in the deep ocean.

**Video Abstract**

**Supplementary Information:**

The online version contains supplementary material available at 10.1186/s40168-022-01263-6.

## Introduction

The deep sea harbors around 75% of the prokaryotic biomass and more than half of the prokaryotic production of the global ocean, and is a key site for organic matter (OM) remineralization and storage in the biosphere [1]. An estimated 1–40% of the photosynthetically fixed carbon in the upper water reach the deep sea [2], which is generally considered oligotrophic in nature [2, 3]. The flux of nutrients varies in intensity and frequency over temporal and spatial scales [4–6], and mass input of particles from surface algal blooms may lead to periodic increases in quantity and bioavailability of OM in the deep ocean [3, 7]. Deep-sea microorganisms therefore have to employ special metabolic strategies to cope with the variable OM conditions to ensure their survival and functioning [3, 7].

Bacteria of the phylum *Chloroflexi* are dominant members of microbial communities in the global deep ocean [8, 9]. For example, the SAR202 clade of the *Chloroflexi* on average accounts for > 10%, and in some cases up to 40% of the total prokaryotic community in meso- and bathypelagic water of the Atlantic and Pacific oceans [8, 10–12]. *Chloroflexi* have also been shown to account for 25.5–41.3% of total 16S rRNA gene sequences in global marine sediments [9, 13, 14]. Currently, the knowledge on the metabolism of deep-sea *Chloroflexi* mainly relies on metagenomic or single-cell genomic analysis, due to the lacking of cultivated representatives for dominant deep-sea lineages [15–17]. These studies revealed that *Chloroflexi* from deep-sea waters harbor genes involved in organosulfur compounds degradation [8, 15], sulfite oxidation [8, 15], and metabolism of recalcitrant compounds such as cyclic alkanes and aromatic compounds [15–17]. The analysis of deep-sea *Chloroflexi* from anoxic subseafloor sediments suggests these bacteria have potential for reductive respiration of organohalogen compounds, and for the fermentation of OM combined with CO_2_ fixation via the Wood-Ljungdahl pathway [9, 18]. These findings suggest that the *Chloroflexi* play important roles in biogeochemical cycles of the deep ocean. However, existing studies only covered a few seawater or anoxic subseafloor sites [8, 9, 15–18]. Given the high heterogeneity of the deep-sea habitats [3] and great phylogenetic and functional diversity of *Chloroflexi* bacteria [9, 17], the current understanding of the metabolisms of deep-sea *Chloroflexi* is therefore incomplete, and their genomic basis and metabolic strategies to adapt to fluctuations of OM supply (e.g., OM with different recalcitrancy) in the deep ocean are unclear.

The hadal trenches, formed by the subduction of tectonic plates, are the deepest part of the ocean [19]. Multiple sources of OM inputs combined with frequent OM remobilization due to special topographies, tectonic activities, and intra-trench currents, lead to higher heterogeneity and fluctuation of OM in the hadal trenches than those in other deep-sea habitats [19–22]. However, despite the complex OM supply and extreme environmental conditions, such as high pressure, active microbial carbon turnover in hadal sediments has been frequently reported, making the hadal trenches “hot spots” of OM remineralization in the deep ocean [23–25]. Recently, *Chloroflexi* have been identified as one of the dominant taxa in seawater and sediment of the hadal trenches [15, 26, 27] and were found to primarily belong to novel lineages [27]. In addition, hadal *Chloroflexi* were not only numerically dominant but also highly transcribed in both hadal seawater and sediments (accounting for up to 36.2% of transcribed prokaryotic 16S rRNA sequences), suggesting high in situ activities [15, 27]. Co-occurrence network analysis further revealed that *Chloroflexi* lineages were important in mediating the interactive network within hadal microbial communities [26, 27]. To date, only three studies reported the metabolism of hadal *Chloroflexi* based on 13 MAGs or single amplified genomes (SAGs) recovered from seawaters [15, 17, 28]. These bacteria were shown to encode enzymes to metabolize chitin, dimethyl sulfoxide, aromatic compounds (e.g., phthalate), and osmolytes [15, 28], but the detailed degradation pathways were unclear. Moreover, although these studies shed light on the lifestyles of hadal *Chloroflexi*, but the limited number of investigated genomes restricted the findings primarily to the pelagic SAR202 group II and III [15, 28]. The metabolic potentials of other dominant and novel lineages of *Chloroflexi* living in the hadal zone are thus largely unknown.

In this study, we employed a metagenomic approach to fill the knowledge gap on the metabolism of *Chloroflexi* that are living in surface sediments of the hadal zone. We obtained unique samples from the deepest point of the ocean, the Challenger Deep of the Mariana Trench. Species composition and activity potential of hadal sediment *Chloroflexi* were revealed using amplicon sequencing of 16S rRNA gene and their transcripts. Representative *Chloroflexi* MAGs were then retrieved from nine metagenomic datasets, and their phylogeny, distribution, and metabolic potentials were explored. All MAGs were found to belong to novel lineages of *Chloroflexi*, representing major and widely distributed members of the hadal sediment microorganisms. The recovered *Chloroflexi* showed the capabilities to degrade a wide range of OM with different levels of recalcitrance. Our analysis also revealed for the first time the presence of hydrolytic and oxidative pathways in deep-sea *Chloroflexi* for the complete degradation of polyaromatic hydrocarbons (PAHs), polychlorobiphenyl (PCBs), and halogenated organic compounds. Potential metabolic strategies to adapt to fluctuation and heterogeneity of OM in the hadal trenches are proposed based on the metabolic reconstruction.

## Results and discussion

### Composition and activity of *Chloroflexi *in sediments of the Challenger Deep

**Fig. 1:**
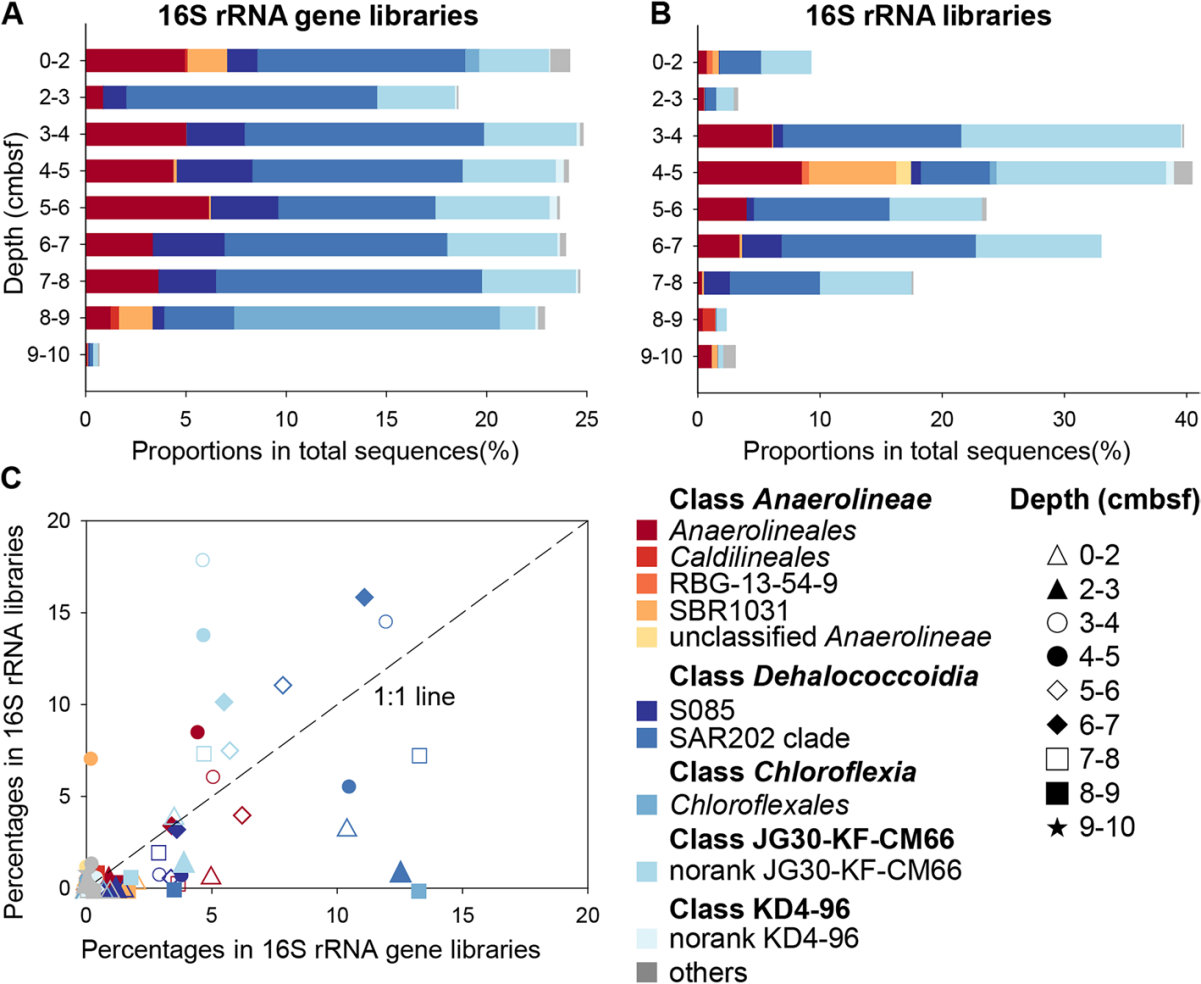
Composition of the bulk (**A**) and potentially active (**B**) *Chloroflexi* in the hadal sediments at the order level, revealed by 16S rRNA gene and 16S rRNA, respectively. The relative activities of different orders are shown as the ratio between their frequencies in the 16S rRNA library and the 16S rRNA gene library at each sediment depth (**C**)

In this study, 16S rRNA genes and their transcripts were sequenced for samples from nine different depths of a sediment core retrieved from the Challenger Deep of the Mariana Trench. The results showed that *Chloroflexi* accounted on average for 20.9% and 19.1% of the total sequences for the bulk (i.e., 16S rRNA gene) and potentially active (i.e., 16S rRNA) bacterial communities, respectively (Fig. 1A, B, and Additional file 1: Fig. S1). The distribution of the bulk *Chloroflexi* population was relatively stable and varied between 18.6 and 24.6% of total rRNA gene sequences in the upper 9 cm (Fig. 1A, Additional file 1: Fig. S1). In contrast, the proportion of the transcribed *Chloroflexi* 16S rRNA sequences varied greatly with depth. *Chloroflexi* transcripts showed the highest proportion at 3–7 cm below seafloor, accounting for up to 40.6% of the total 16S rRNA sequences (Fig. 1b, Additional file 1: Fig. S1). The bulk and potentially active *Chloroflexi* populations were mainly composed of members from classes *Anaerolineae*, *Dehalococcoidia*, *Chloroflexia*, JG-KF-CM66, and KD4-96, among which *Anaerolineae*, *Dehalococcoidia*, and JG-KF-CM66 were the most dominant and highly transcribed ones (Fig. 1B and C). These results are consistent with previous findings on the microbial composition of hadal trench sediments [26, 27], indicating the general significance of *Chloroflexi* in maintaining the structure and functions of the hadal biosphere. Compared with deeper sediment layers, *Chloroflexi* showed relatively lower transcription levels at 0–3 cm, and the possible reasons include (1) The physical/chemical conditions (such as high oxygen levels and organic carbon content) of the surface layers might favor the transcriptional activities of other bacterial taxa, leading to lowered proportions of *Chloroflexi* in the transcript pool [23, 25]; (2) A fraction of the microbes at these layers might be sourced from shallower habitats such as sediment of the trench slopes due to lateral transportation [19, 21]. These microbes may show low transcriptional activity in hadal trench sediments, due to the extreme environmental conditions, such as high pressure; (3) the potentially higher level of degradation of RNA in the surface layer sediments during sample recovery from the deep trench [27].

### MAG reconstruction, genome description, and phylogenomic analysis

A total of 62 *Chloroflexi* MAGs with completeness > 50% and contamination < 5% were reconstructed from the nine metagenomes covering different sediment layers (Additional file 2: Table S1). These MAGs were further dereplicated at 99% average nucleotide identity (ANI) to yield 17 representatives with an average completeness of 68.56% (51.38–92.99%) and contamination ranged from 0.00 to 3.64% (Table 1 and Additional file 2: Table S1). The genome sizes were estimated to range between 1.85 and 3.90 Mbp, and GC contents were between 58.64 and 69.45% (Table 1). Currently, only thirteen genomes of *Chloroflexi* have previously been reported from the hadal zone (deeper than 6000 m), and 11 of them were from seawater [15, 17, 28]. Only 2 *Chloroflexi* MAGs (GCA_004356475.1 and GCA_004356815.1) found in the NCBI database were recovered from hadal sediments, but without any description on their metabolism.

**Table 1:** Summary of the 17 representative MAGs retrieved from sediments of the Challenger Deep

MAGs	Completeness (%)	Contamination (%)	Contig no.	GC%	CDS no.	Estimated genome size (Mbp)	Sequencing depth
MT2_13^a^	92.99	0.00	275	58.79	2237	2.74	21×
MT4_27	89.96	0.31	215	65.69	1934	2.05	41×
MT6_15	87.27	3.64	168	58.64	2397	2.85	28×
MT4_14	86.30	1.98	408	60.36	2572	3.05	25×
MT2_3	85.70	0.00	472	59.61	3150	3.90	24×
MT6_13	84.77	0.11	425	60.34	2430	2.99	29×
MT1_55	73.57	0.00	386	59.26	1683	2.33	33×
MT5_44	67.62	1.19	314	65.58	1512	2.29	14×
MT5_40	66.01	0.20	359	58.76	1610	2.61	18×
MT1_49	60.51	3.08	400	62.83	2008	3.18	21×
MT1_63	56.40	2.97	296	59.82	1319	2.46	15×
MT4_29	54.89	2.18	365	62.58	1770	3.09	15×
MT8_34	52.62	0.00	467	59.67	1199	2.16	22×
MT2_40	52.07	2.20	253	69.45	1108	2.07	25×
MT6_44	51.99	0.00	486	59.57	1031	1.85	23×
MT9_49	51.53	2.38	257	69.22	1189	2.16	26×
MT1_74	51.38	0.61	255	65.29	1016	1.98	18×

^a^MAGs were named using “site + layer + genome number”, for example MT2_13 means the 13th genome from sediment of 2–3 cm below seafloor from the Mariana Trench

**Fig. 2:**
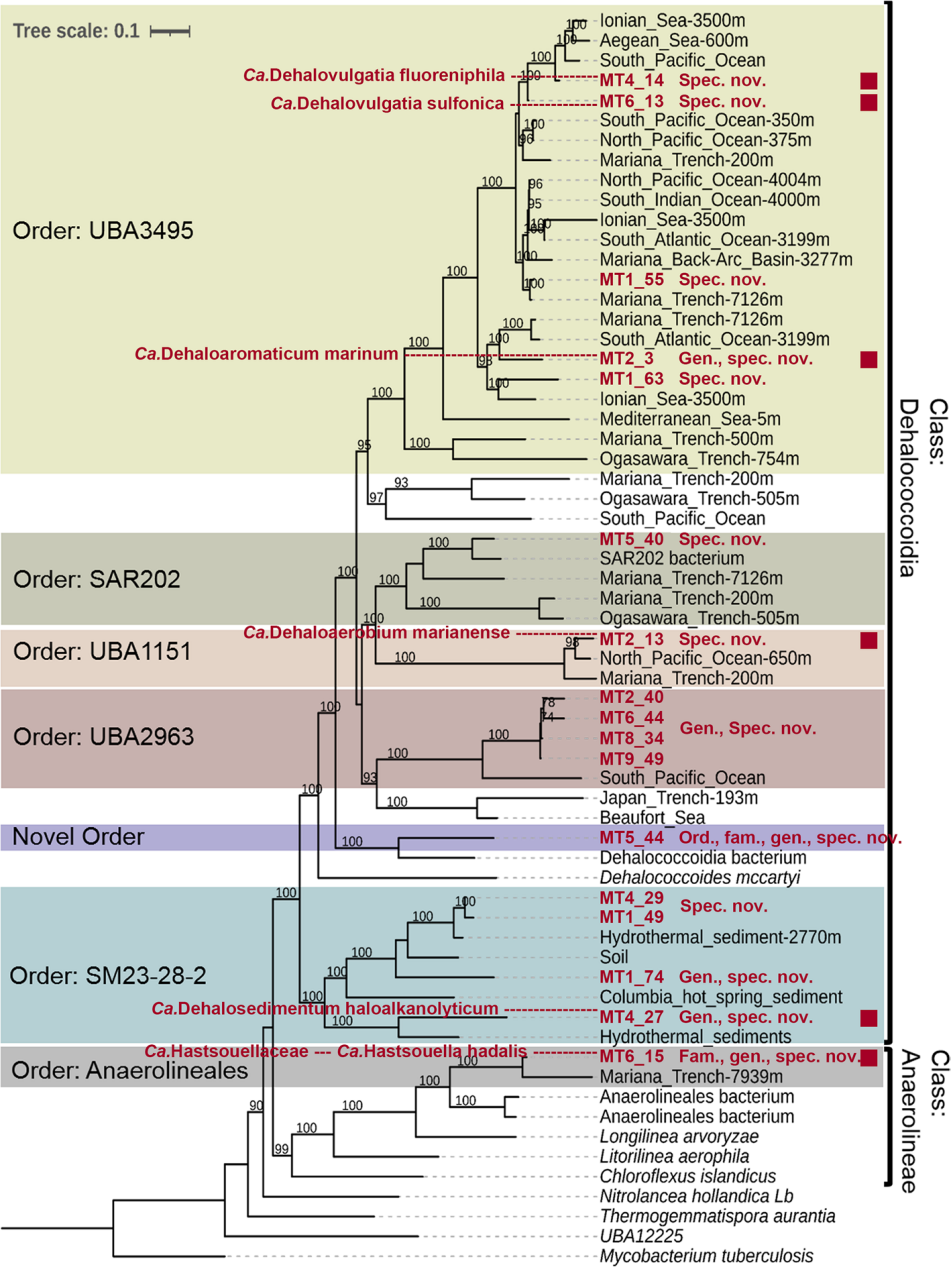
Maximum likelihood phylogenomic tree of the 17 selected *Chloroflexi* MAGs. Genome of *Mycobacterium tuberculosis* was used as the root. Bootstrap values were calculated based on 100 replicates and the values higher than 90% were indicated at the base of the corresponding node. The colored backgrounds show the genomes belonging to the same order. The taxonomy was determined using GTDB-tk and the novelty of the recovered genomes was determined based on GTDB classification. Red square indicates the MAGs with completeness > 80%

Phylogenomic analysis showed that the retrieved MAGs belonged to the classes *Anaerolineae*, *Dehalococcoidia*, and SAR202 (previously classified as a class) (Fig. 2, Additional file 2: Table S2, Additional file 1: Fig. S2). Taxonomic classification was further conducted with GTDB-Tk toolkits [29] (Fig. 2 and Additional file 2: Table S3). The results revealed that these MAGs represent six novel species (MT1_49, MT2_13, MT5_40, MT1_63, MT1_55, MT4_14; relative evolutionary divergence, RED, ranged from 0.89 to 0.99) and four novel genera (MT4_27, MT1_74, MT9_49, MT2_3; RED ranged from 0.76 to 0.89) in the orders SM23-28-2, SAR202 (formerly SAR202 group II), UBA2963 (formerly SAR202 group VII), UBA1151(formerly SAR202 group I), and UBA3495 (formerly SAR202 group III) of the class *Dehalococcoidia* (Fig. 2, Additional file 2: Table S3 and Additional file 1: Fig. S2). In addition, MT5_44 (RED = 0.52) represents a novel order in the class *Dehalococcoidia*, and MT6_15 (RED = 0.67) represents a novel family in the order *Anaerolineales* of the class *Anaerolineae* (Fig. 2 and Additional file 2: Table S3).

Six MAGs, i.e., MT6_15, MT4_27, MT2_13, MT2_3, MT6_13, and MT4_14, showed completeness higher than 80% and contamination lower than 3.6% (Table 1), and are therefore qualified as type materials according to the criteria defined recently for the taxonomy of uncultivated prokaryotes [30, 31]. Following the guidelines developed by Genomics Standards Consortium [32], Konstantinidis et al. [33], and Chuvochina et al. [34], we propose the names *Candidatus* Hastsouellaceae (fam. nov.) and *Ca.* Hastsouella hadalis (genus nov. and species nov.) for MT6_15, *Ca.* Dehalosedimentum haloalkanolyticum (genus nov. and species nov.) for MT 4_27, *Ca.* Dehaloaerobium marianense (species nov.) for MT2_13, *Ca.* Dehaloaromaticum marinum (genus nov., species nov.) for MT2_3, *Ca.* Dehalovulgatia sulfonica (species nov.) for MT6_13, and *Ca.* Dehalovulgatia fluoreniphila (species nov.) for MT4_14. Explanations on nomenclature can be found in Additional file 3, and protologues accompanying the proposed names can be found in Table S4.

### Distribution of the reconstructed MAGs in hadal sediments and other ecosystems

**Fig. 3:**
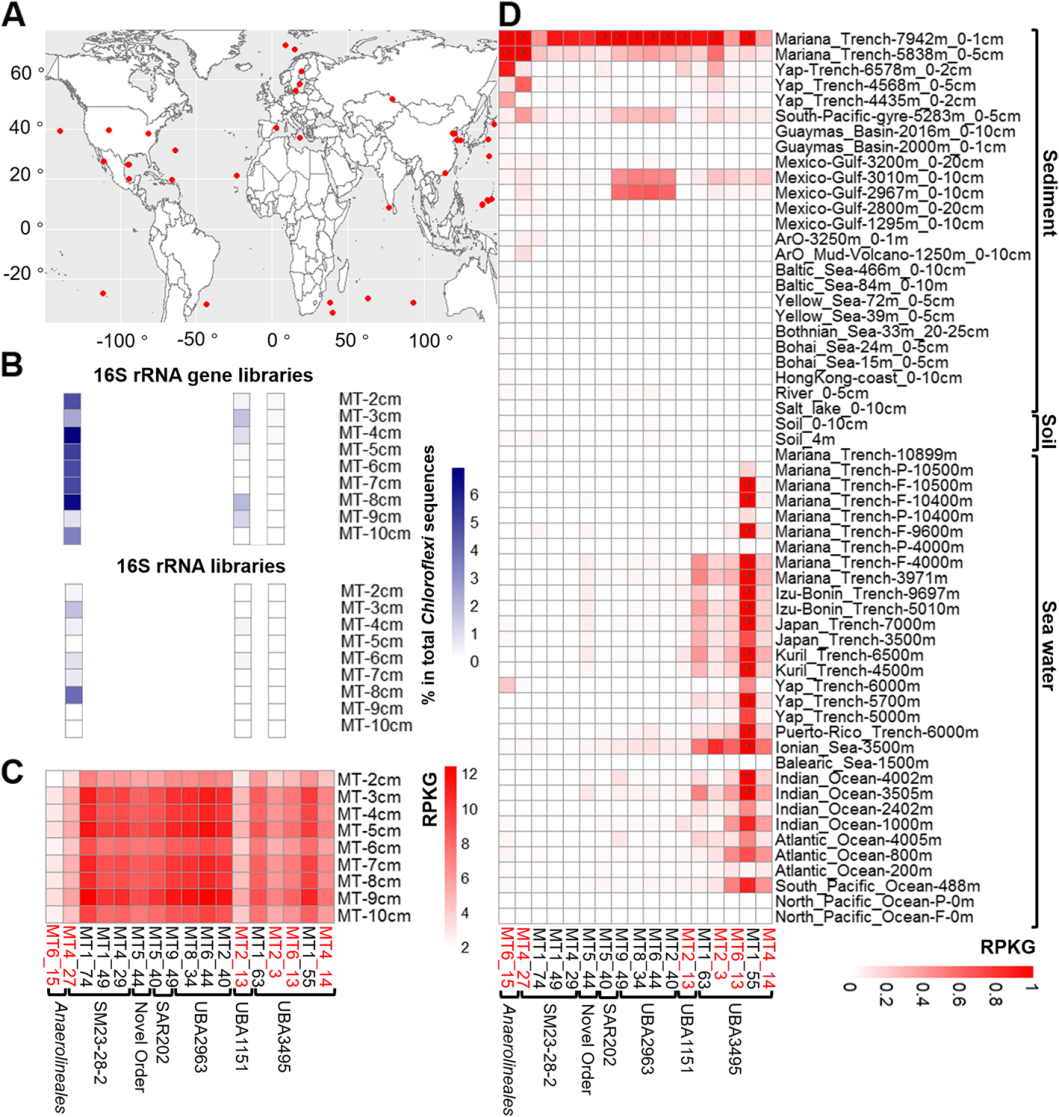
Distribution of the recovered MAGs in hadal sediments and other natural ecosystems. (**A**) The sampling sites of the datasets included in the analysis; (**B**) the relative abundance of the closest matched OTUs in 16S rRNA and 16S rRNA gene libraries of sediments of the Challenger Deep. The MAGs without any value in 16S rRNA or 16S rRNA gene libraries mean no matched OTUs due to a lack of 16S rRNA gene in the corresponding MAG; (**C**) the reads recruitment of the recovered MAGs in metagenomes from different layers of hadal sediments of the Challenger Deep; and (**D**) reads recruitments in other natural ecosystems. The names of samples from sediments were shown as “sampling site (region)_water depth of the site_depth below seafloor.” The black dots in the heatmap boxes indicate outlier values. Red-colored names at the bottom indicate the MAGs with completeness > 80%

Among the 17 representative MAGs, only MT4_27 was found to contain partial 16S rRNA gene. We further searched all of the 62 MAGs recovered, and found 2 additional MAGs that contain partial 16S rRNA gene, i.e., MT9_9 (represented by MT2_13) and MT8_3 (represented by MT2_3) (Additional file 2: Table S1). These 16S rRNA gene sequences matched with three OTUs from 16S rRNA and 16S rRNA gene libraries constructed in this study (Fig. 3B). The matched OTUs were mainly distributed in the upper 8 cm of the sediment core and together accounted for 4.2–8.5% and 0.1–4.0% of *Chloroflexi* sequences in the 16S rRNA gene and 16S rRNA libraries, respectively (Fig. 3B). Recruitment of shotgun sequencing reads showed that the 17 representative MAGs were present in all depths (0–10 cm below seafloor) of the sediment core, with MT6_44, MT1_74, MT8_34, MT9_49, and MT2_40 being the most abundant (Fig. 3C). The three MAGs with 16S rRNA gene showed the lowest recruitment values (Fig. 3C). It is therefore reasonable to postulate that the 14 MAGs without 16S rRNA genes might be more abundant in the bulk and active bacterial communities than the three MAGs with 16S rRNA gene (Fig. 3B). These results suggest that the recovered MAGs represent major members of *Chloroflexi* in the hadal sediment of the Mariana Trench.

The global distribution of the recovered MAGs was evaluated by read recruitments against 58 publicly available metagenomes derived from different natural habitats, including seawater and surface sediments from different depths of the open ocean, sediments of mud volcanos, deep-sea oil spilling sites, deep subseafloor, coastal regions, rivers, and salt-lakes, as well as soils (Fig. 3A, D and Additional file 2: Table S5). All of the 17 MAGs showed the highest recruitment values in surface sediments of the Mariana Trench, including the nine samples analyzed in this study (water depth of 10853 m) (Fig. 3C) and two previous samples with depths of 7942 m and 5838m (Fig. 3D and Additional file 2: Table S5), which likely reflect the biogeographic distributions impacted by local environmental selection [8]. The majority of the MAGs (except the order SM23-28-2) have recruited reads from metagenomes derived from sediments and seawaters of the global deep ocean (Fig. 3D and Additional file 2: Table S5), and none of the MAGs was present in seawater or sediments from shallow habitats, including the epi-pelagic zone of the open ocean, coastal regions, river, salt-lake, or soil (Fig. 3D). The results suggest that majority of the recovered MAGs are widespread in seawater and surface sediment of the deep ocean.

However, MAGs from different orders showed apparent preferences on their distributions in different deep-sea habitats. MAGs from the order UBA3495 (formerly SAR202 group III) showed high recruitment values in metagenomes of both deep seawater and sediment (Fig. 3D). The SAR202 group III has previously been shown to be one of the most dominant *Chloroflexi* in the water column of the global deep ocean [17, 28], and our results highlight the significance of these bacteria in both pelagic and sedimentary habitats of the deep ocean. Expansion of paralogous enzymes, such as flavin-dependent monooxygenases, in SAR202 group III has been suggested to be important for their adaptation to different deep-sea habitats, by diversifying the range of organic molecules that the cells can utilize [16, 17]. In contrast to UBA3495, MAGs from the orders *Anaerolineales*, SAR202 (formerly SAR202 group II), UBA2963 (former SAR202 group VII), UBA1151(formerly SAR202 group I), and the novel order (MT5_44) showed higher recruit values in metagenomes from deep-sea sediments compared to those from seawater, suggesting their preferential distribution in deep-sea sediment habitats. Interestingly, the MAGs of the order SM23-28-2 (particularly MT1_74) only matched with the reads from sediment metagenomes of the Mariana Trench (Fig. 3D), indicating a potential endemism to the Mariana Trench, which might be a result of long-term adaptation to the special geographic, physical and chemical conditions of the Mariana Trench, such as extreme hydrostatic pressure, tectonic activity, geographic isolation, and nutrient inputs [19, 35].

### Metabolic potential for degradation of carbohydrates, fatty acids, proteins, and organosulfur compounds

**Fig. 4:**
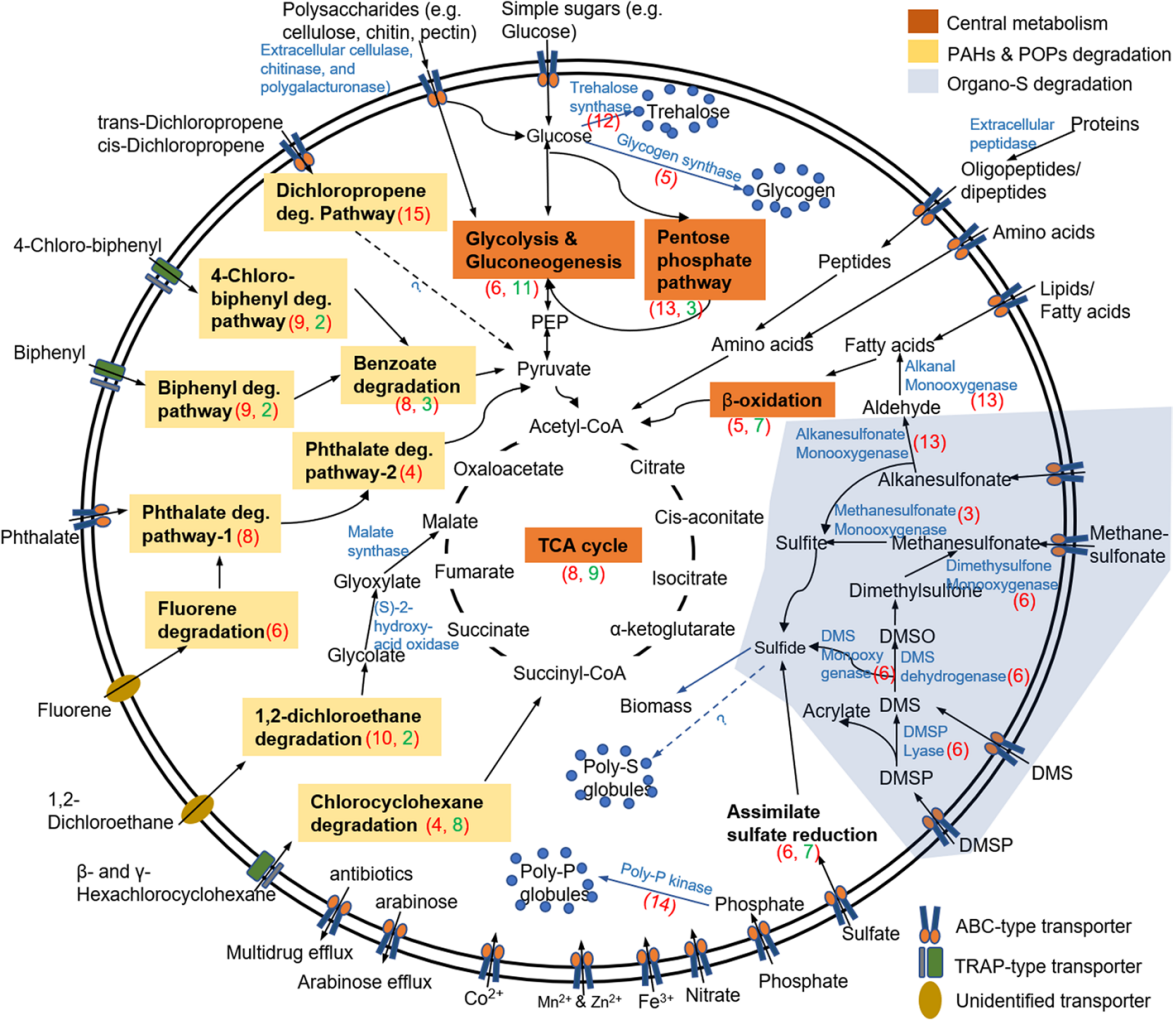
Overview of the metabolic potentials in the 17 assembled *Chloroflexi* MAGs. Black arrows show the annotated metabolic pathways and the linkage between different metabolic flows. The pathways with light blue background show the degradation pathways of organosulfur compounds, and those with yellow background show the degradation of different recalcitrant compounds. The red and green values in brackets are numbers of MAGs that encode complete and partial pathways/enzymes, respectively. If only a red value is shown, the associated pathway/enzyme is complete in all of the related MAGs

Considering the varied completeness (51.38–92.99%) of the retrieved MAGs, the downstream functional analysis was mainly focused on genes and pathways that were successfully identified and annotated from the MAGs. Discussion on absent genes/pathways was avoided to eliminate possible misleading conclusions due to the incompleteness of the genomes. Genome annotation of the recovered MAGs revealed their potentials for organo-heterotrophic metabolisms and utilization of a wide range of OM (Fig. 4). Gene sets encoding for complete/near-complete pathways or key enzymes in the central carbohydrate metabolism, including glycolysis, tricarboxylic acid cycle (TCA cycle), pentose phosphate pathway, and β-oxidation of fatty acids, were present in all MAGs with genome completeness > 80% (Fig. 4 and Additional file 2: Table S6). These pathways allow the degradation/transformation of simple sugars (e.g., glucose), fatty acids, as well as amino acids. On top of this, genes encoding extracellular cellulases (MT4_29, MT2_13, MT1_55), chitinases (occurred in most MAGs), and polygalacturonases (MT2_13) as well as ABC-type transporters for polysaccharides were also present in the MAGs (Fig. 4 and Additional file 2: Table S7), suggesting the potential to degrade complex polysaccharides, such as cellulose, chitin or pectin. In addition, different types of peptidases as well as ABC-type transporters for amino acids, dipeptides and oligopeptides were found to be present in the *Chloroflexi* MAGs, indicating their potential to degrade protein detritus (Fig. 4 and Additional file 2: Table S7).

**Fig. 5:**
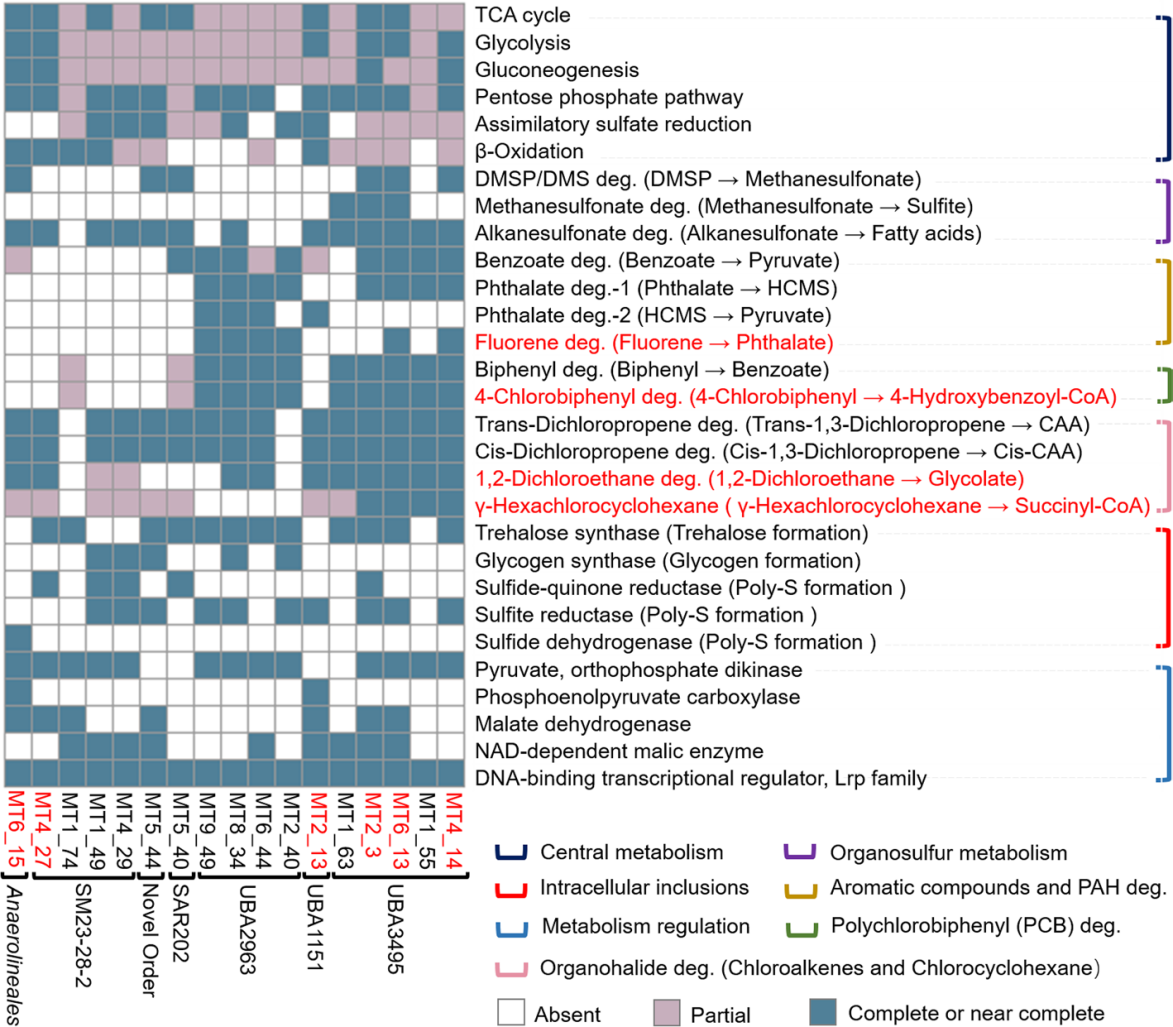
Completeness of the metabolic pathways identified in the *Chloroflexi* MAGs. “Complete or near complete” indicates pathways that were complete or with one enzyme missing, “partial” indicates pathways with two or more enzymes missing, and “absent” means none of the enzymes in the pathways were identified. The red-colored pathways were further illustrated in Fig. 6 for detailed reaction flows. Red-colored names at the bottom indicate the MAGs with completeness > 80%

The hadal sediment *Chloroflexi* MAGs also had the potential capability to degrade various organosulfur compounds (Fig. 4). Alkanesulfonate monooxygenase coding genes were present in 13 of the 17 recovered MAGs (Figs. 4 and 5 and Additional file 2: Table S7). This enzyme cleaves the carbon-sulfur bonds in a wide range of sulfonated alkanes to produce sulfite and aldehyde [36], with the latter being oxidized to fatty acid by an alkanal monooxygenase, whose coding gene was also present in 13 of the 17 MAGs (Figs. 4 and 5 and Additional file 2: Table S7). In addition, genes encoding homologs of enzymes involved in dimethylsulfide (DMS) (i.e., DMS monooxygenase and DMS dehydrogenase) and methanesulfonate metabolisms (i.e., methanesulfonate monooxygenase) were found in the MAGs (Figs. 4 and 5), and genes encoding the ABC-type sulfonate transporters were also identified (Fig. 4 and Additional file 2: Table S7). These results suggested the potential of hadal sediment *Anaerolineae* and *Dehalococcoidia* to utilize multiple organic sulfur compounds as energy, carbon and sulfur sources, a finding that is similar with previous reports on SAR202 clade (primarily SAR202 group III) from deep seawater [8, 28].

### Pathways for the degradation of phthalate, benzoate, polyaromatic hydrocarbons and polychlorobiphenyl compounds

**Fig. 6:**
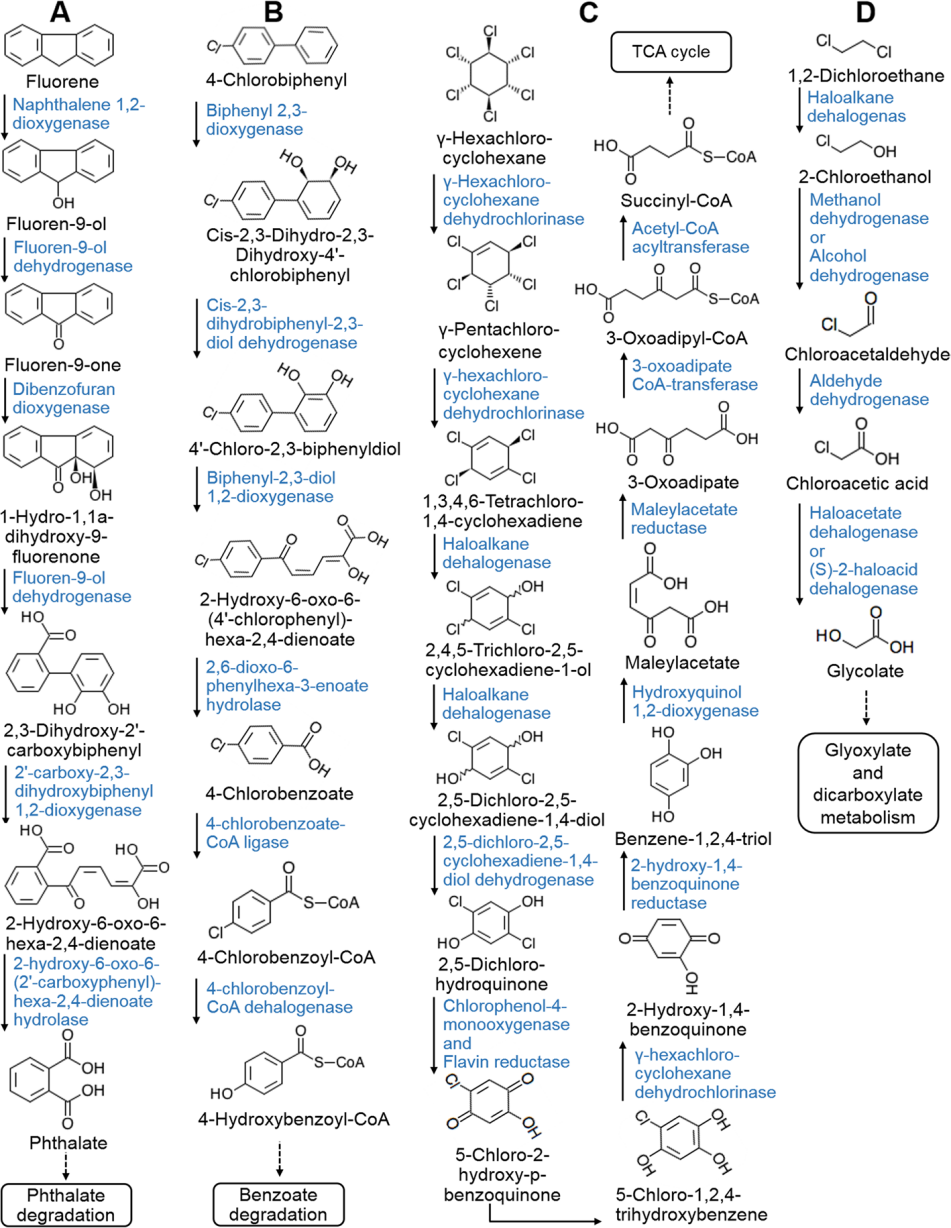
The degradation pathways of representative PAHs and POPs identified in hadal sedimentary *Chloroflexi* MAGs. These pathways were potentially utilized for complete degradation of (**A**) fluorene, (**B**) 4-chlorobiphenyl, (**C**) 1,2-dichloroethane, and (**D**) γ-hexachlorocyclohexane. The illustrated pathways were found to be complete in at least one MAG recovered in this study

The hadal *Chloroflexi* MAGs harbored pathways for the degradation of benzoate and phthalate (Figs. 4 and 5). Eight MAGs from the orders SAR202, UBA2963 and UBA3495 contained complete or near complete gene clusters encoding six enzymes for the degradation of benzoate to pyruvate or oxaloacetate (Figs. 4 and 5, Additional file 2: Table S7 and Additional file 1: Fig. S3). Eight MAGs from the orders UBA2963 and UBA3495 contained genes encoding complete or near complete pathways for degradation of phthalate to 4-carboxy-2-hydroxymuconate semialdehyde (HCMS) (Figs. 4 and 5, Additional file 2: Table S7 and Additional file 1: Fig. S4), and four MAGs from UBA2963 and UBA1151 contained genes encoding enzymes that can further degrade HCMS to pyruvate (Fig. 5, Additional file 2: Table S7 and Additional file 1: Fig. S4). As benzoate and phthalate are common intermediates in the degradation of many aromatic compounds, we hypothesized that the recovered MAGs might also be able to degrade substrates with more complex structures. Indeed, complete or near complete pathways for the degradation of polyaromatic hydrocarbons (e.g., fluorene) and polychlorobiphenyls (PCBs, e.g., biphenyl and 4-chlorobiphenyl) were found (Figs. 4 and 5). Six MAGs from the orders UBA2963 and UBA3495 harbored near complete pathways for the transformation of fluorene to phthalate (Figs. 5 and 6 and Additional file 2: Table S7). Nine MAGs from the orders UBA2963 and UBA3495 harbored complete or near complete pathways for the transformation of biphenyl to benzoate (Figs. 4 and 5, Additional file 2: Table S7 and Additional file 1: Fig. S5). In addition, the nine MAGs from the orders UBA2963 and UBA3495 also contained complete or near complete pathways for the degradation of 4-chlorobiphenyl to 4-hydroxy-benzoyl-CoA (Figs. 5 and 6 and Additional file 2: Table S7), which can be further metabolized via benzoate degradation pathway (Fig. 6).

As labile OM is usually readily utilized by microorganisms in the upper water layers, the remaining OM in the deep ocean often includes a variety of structurally complex compounds, such as aromatic compounds [37]. Partial pathways of phthalate degradation (phthalate to protocatechuate), and some enzymes involved in the degradation of benzoate (e.g., catechol 2,3-dioxygeenase) have been reported in SAR202 MAGs/SAGs from seawater of hadal trenches and other deep-sea environments [15–17, 28], and related genes were highly transcribed in situ [15, 28]. Our study advances the existing knowledge by identifying the complete pathways for the degradation of phthalate and benzoate to CO_2_ by hadal sediment *Chloroflexi*, and is the first time to show that deep-sea *Chloroflexi* harbor pathways to completely degrade fluorene, biphenyl and 4- chlorobiphenyl.

### Pathways for hydrolytic degradation of halogenated organic compounds

In this study, we further discovered in hadal sediment *Chloroflexi* the prevalence of genes encoding haloalkane dehalogenase, haloacetate dehalogenase, and 2-haloacid dehalogenase (Fig. 5 and Additional file 2: Table S7), which catabolize hydrolytic dehalogenation, replacing the halogen atoms in organohalides with hydroxyl groups [38]. These enzymes have a broad specificity and participate in the degradation of multiple halogenated OM [38]. Complete or near-complete pathways for the hydrolytic and oxidative degradation of several chloroalkenes and chlorocyclohexane were further revealed (Figs. 4 and 5). Ten MAGs from the orders *Anaerolineales*, SM23-28-2, UBA2963, UBA1151, and UBA3495 harbored genes encoding the complete or near-complete pathways for the degradation of 1,2-dichloroethane to glycolate (Figs. 5 and 6 and Additional file 2: Table S7), which can either be further transformed and enter the TCA cycle or be utilized for vitamin B6 biosynthesis. The same pathway also catabolizes the degradation of *trans*-dichloropropene and *cis*-dichloropropene to *trans*-3- and *cis*-3-chloroacrylic acid, respectively (Figs. 4 and 5, Additional file 2: Table S7 and Additional file 1: Fig. S6). In addition, a pathway for the complete degradation of γ-hexachlorocyclohexane to succinyl-CoA (an intermediate in the TCA cycle) was reconstructed in the *Chloroflexi* MAGs (Figs. 4 and 5). The entire pathway involves 11 enzymes (Fig. 6 and Additional file 2: Table S7), and complete or near complete sets of genes encoding these enzymes were present in four MAGs from the order UBA3495 (Fig. 5). Eight MAGs from orders *Anaerolineales*, SM23-28-2, SAR202, UBA1151, UBA3495 and the novel order (MT5_44) also encode for the majority of enzymes for γ-hexachlorocyclohexane degradation, but with 2-6 enzymes missing (Fig. 5 and Additional file 2: Table S7).

Currently, deep-sea *Chloroflexi* have mainly been implied in reductive dehalogenation [9, 39, 40], a strictly anaerobic process which utilizes halogenated organic compounds as electron acceptor to oxidize hydrogen (or formate) [41]. In contrast, the hydrolytic and oxidative degradation of organohalides are aerobic processes [38]. The genes coding for haloalkane and haloacetate dehalogenases have been previously reported in *Chloroflexi* genomes from oxic abyssal sediments [42], but our study revealed for the first time the complete or near complete pathways for hydrolytic and oxidative degradation of multiple types of organohalides in hadal *Chloroflexi* (Figs. 4 and 5). The MAGs in this study were recovered from surface sediments of the Challenger Deep at depth of 0–10 cm below seafloor, which were well oxygenated as revealed by in situ oxygen measurements conducted at the same site [25]. Such an environmental condition well supports the potential of the studied *Chloroflexi* to degrade organohalides via the annotated pathways. In addition, reads recruitment showed that the majority of the MAGs retrieved in this study were also widely distributed in global deep-sea water and surface sediment (Fig. 3), which highlights the significance of *Chloroflexi* in carbon and halogen cycling in oxygenic habitats of the deep ocean.

### Microbial degradation of persistent organic pollutants in the deepest ocean

The metabolic reconstruction of the recovered MAGs in this study revealed the potential of hadal sediment *Chloroflexi* for the complete degradation of different types of recalcitrant organic compounds, including PAHs (e.g., fluorene), PCBs (e.g., 4-chlorobiphenyl), haloalkanes (e.g., 1,2-dichloroethane and 1,3-dichloropropene), and chlorocyclohexane (e.g., γ-hexachlorocyclohexane) (Fig. 5 and Additional file 2: Table S7). These findings have important implications for the deep ocean ecosystems, in general, and the hadal trench systems in particular. Many of these compounds are listed as persistent organic pollutants (POPs) by the Stockholm Convention [43] and their presence and accumulation in deep-sea organisms and environments have been widely reported [44, 45]. Recent studies have further revealed that PCBs, microplastics, heavy metals, and halogenated organic pollutants have even accumulated in the deepest trenches of the ocean [46–49], suggesting that the anthropogenic pollutants can be an important part of the OM pool in the hadal trenches. Many types of PAHs and POPs-like compounds can however also be naturally produced via biotic (e.g., biosynthesis via halogenase or haloperoxidase) and abiotic processes (e.g., peroxidative mechanisms, photochemical reactions, volcanic activities) [39, 50, 51], and can be enriched in the deep ocean via the “biological pump” [52]. The capability to metabolize these recalcitrant OM would likely provide *Chloroflexi* bacteria with survival advantages in nutrient/energy-limited habitats, which might be one of the reasons for their dominance in the sediments of the hadal trenches as observed in this (Fig. 3) and previous studies [26, 27]. On the other hand, close interactive networks have been reported to occur between *Chloroflexi* lineages and between *Chloroflexi* and other microbial taxa in the hadal trench sediments [27], which supports the possibility of co-metabolism during the degradation of recalcitrant OM [53]. For example, the degradation of halogenated organic matter by *Chloroflexi* (i.e., dehalogenation process) may produce semi-labile intermediates serving as substrates for other taxa in the microbial community [41]. The capability to degrade recalcitrant OM and the potential co-metabolism relationships with other microbial taxa might be one of the reasons for previous observations that *Chloroflexi* lineages play keystone roles in interactive networks of the microbial community in hadal trench sediments [27].

### A potentially “feast-or-famine” metabolic strategy in response to fluctuating supply of OM

**Fig. 7:**
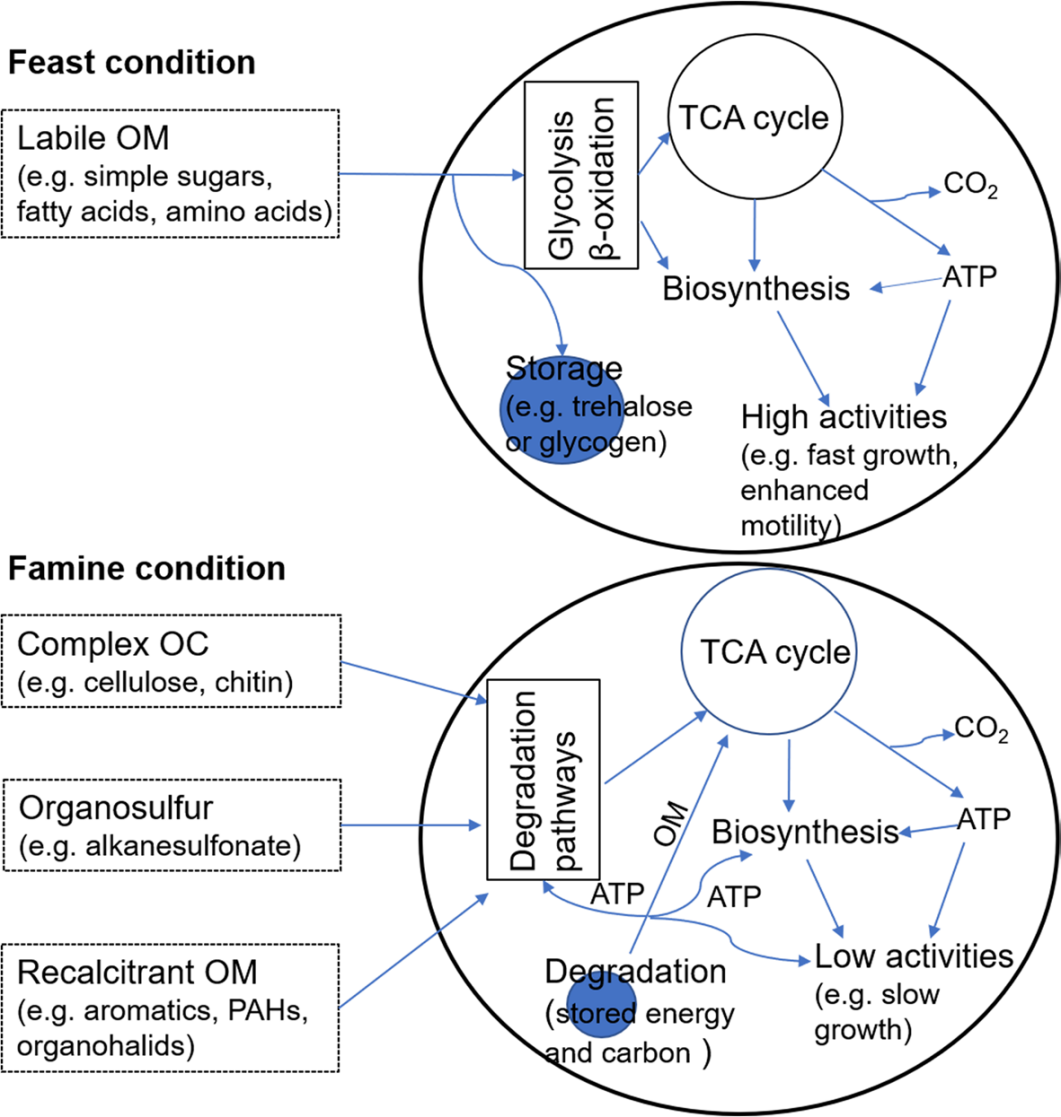
The proposed “feast-or-famine” metabolic strategy for hadal sediment *Chloroflexi* recovered in this study

Deep-sea benthic communities experience feast-or-famine conditions due to the periodical and spatial variations in the input of particles in a generally energy-depleted environment [7]. Deep-sea microbial communities have been shown to respond rapidly to nutrient input, even after long periods of starvation [54]. However, little is known about the genomic basis and potential metabolic strategies of deep-sea microorganisms for such a lifestyle. Our results showed that the hadal *Chloroflexi* exhibited capabilities of degrading a wide range of organic carbon, sulfur, and halogen compounds (Figs. 4 and 5), including not only labile OM, but also many types of recalcitrant organic compounds (Figs. 5 and 6). In addition, the majority of the MAGs harbor genes encoding alpha, alpha-trehalose synthase, trehalose-6-phosphate synthase and trehalose-phosphate-phosphatase (Fig. 5 and Additional file 2: Table S7), which are key enzymes for the formation of trehalose, a type of intracellular energy storage compound [55]. Some of the MAGs also harbored genes encoding starch (glycogen) synthase and 1,4-alpha-glucan branching enzyme involved in the biosynthesis of glycogen (Fig. 5 and Additional file 2: Table S7) [56], polyphosphate kinase, and phosphate transporter proteins involved in the formation of polyphosphate inclusions (Additional file 2: Table S7) [57], or sulfide-quinone reductase, sulfite reductase, and sulfide dehydrogenase involved in the formation of sulfur globules (Fig. 5 and Additional file 2: Table S7) [56]. Such features are consistent with a “feast-or-famine” metabolic strategy (Fig. 7). During the “feast” condition, such as a pulse input of particulate OM due to diatom bloom in the surface water [7], the bacteria might preferentially uptake and consume labile OM, and excess energy, carbon, and other elements can be stored as intracellular inclusions (Fig. 7). During “famine” condition (i.e., nutrient depleted), the bacteria may enter the “famine” mode to acquire energy from the stored inclusions, and/or from degrading the recalcitrant OM available in the surrounding environments (Fig. 7).

In support of such a “feast-or-famine” lifestyle, the MAGs also encode modules for regulating metabolism in response to changes in nutrient conditions (Fig. 5). The majority of the MAGs contained genes encoding pyruvate orthophosphate dikinase, malate dehydrogenase, and malic enzyme (Fig. 5), and two of the MAGs also showed potential to encode PEP carboxylase (Fig. 5). These enzymes catalyze the inter-conversions between pyruvate, PEP, oxaloacetate, and malate (Additional file 1: Fig. S7), which interconnect the central carbon metabolic pathways (e.g., the TCA cycle and biosynthesis) and are responsible for regulating carbon fluxes among catabolism, anabolism, and energy supply according to the physiological conditions of the cell (Additional file 1: Fig. S7) [58]. In addition, all of the recovered MAGs harbored the *lrp* gene (COG1522) encoding the leucine-responsive regulatory protein (LRP) (Fig. 5), which is one of the “feast-or-famine” regulatory proteins that control the expression of more than 30% of bacterial genes in response to changes in nutrient levels [59, 60]. The existence of these regulatory genes suggests the potential of the hadal *Chloroflexi* to rapidly change the metabolism and physiology under the feast or famine conditions, although the detailed regulation mechanisms can be very complex and are still unknown. Similarly, previous researchers have shown that common marine bacteria (e.g., *Vibrio*, *Alteromonas*, *Colwellia*) from surface seawaters also followed “feast-or-famine” strategy which allows these bacteria to respond rapidly to the changes in nutrient conditions [61]. These findings suggest that the “feast-or-famine” lifestyle might be a common strategy for marine microorganisms to adapt to variable nutrient conditions.

In addition to the changes in nutrient conditions, other environmental factors such as oxygen levels might also trigger the shift between feast and famine metabolic modes of *Chloroflexi* in surface sediments. For example, input of fresh particles from surface water algal blooms [7, 62] may greatly stimulate the respiration and growth of microorganisms in surface hadal sediment, leading to rapid depletion of dissolved oxygen and the shift from aerobic to anaerobic condition. According to the metabolic potentials annotated (Figs. 4 and 5), the hadal *Chloroflexi* may consume labile organic carbon (such as simple sugars, amino acids) and store the excess energy intracellularly (e.g., in the form of trehalose, glycogen or poly-P) under aerobic condition and degrade the intracellular energy storage compounds for energy generation under subsequent anaerobic condition. In fact, oxygen-triggered “feast-or-famine” metabolic strategy has been well documented in polyphosphate accumulating organisms (PAOs) from wastewater treatment systems [63, 64], although the detailed mechanisms might be different from those in hadal *Chloroflexi* (e.g., PAOs usually synthesize polyhydroxyalkanoate under anaerobic condition but no related genes were found in *Chloroflexi* MAGs).

## Conclusions

This study provides an extensive exploration of the metabolic potential of novel and dominant *Chloroflexi* lineages retrieved from the hadal sediments of the Mariana Trench. The results demonstrated a high metabolic plasticity of the hadal sediment *Chloroflexi*, including the complete pathways for hydrolytic or oxidative degradation of recalcitrant OM such as PAHs, PCBs, and organohalides. Our findings expand the current understanding on metabolic capabilities of deep-sea *Chloroflexi*, and highlight their significance on carbon, sulfur, and halogen cycling in the deep ocean. The metabolic plasticity, the capability to form intracellular storage inclusions, as well as the regulatory modules to respond to nutrient conditions discovered in the MAGs support the notion that the hadal sediment *Chloroflexi* employ a “feast-or-famine” metabolic strategy. Such a metabolic strategy allows the bacteria to fulfill energy and nutrient requirement via degradation of different substrates according to the nutrient conditions, and regulate the cell activities (e.g., growth, motility) accordingly, providing advantages for their adaptation to the variable OM conditions in the hadal trenches and other deep-sea habitats. This study therefore provides new perspectives on the metabolism and adaptation strategies of *Chloroflexi* in deep-sea environments.

## Material and methods

### Site description and sampling

Sediment samples were obtained from the Challenger Deep of the Mariana Trench (site MT, 11.4037 °N, 142.3630 °E, water depth of 10853 m) during the cruise from December 2016 to January 2017 by the MV *Zhangjian*. Samples were collected using a box corer attached to a Hadal Lander [25]. Details of the sampling procedure were given in Liu et al. [27]. After recovering onboard, the sediment samples were immediately subsampled using sterile plastic corers and stored at – 80 °C on board.

### Amplicon sequencing analysis on 16S rRNA and rRNA gene diversity

Sediment cores were thawed on ice and were depth fractioned to 0–2-, 2–3-, 3–4-, 4–5-, 5–6-, 6–7-, 7–8-, 8–9- and 9–10-cm subsamples. Total DNA and RNA were co-extracted from triplicate 1-g sediments of each depth fraction using the PowerSoil Total RNA Isolation Kit and DNA Elution Accessory Kit (MoBio Lab, USA) following the manufacturer’s instructions. The RNA samples were treated with DNase I and cDNAs were synthesized using the GoScriptTM Reverse Transcription System (Promega, USA) with random primers. DNA and cDNA samples were amplified with a barcoded primer set 338F/806R targeting the V3–V4 hypervariable regions of bacteria [65]. Detailed procedure for DNA/RNA coextraction and PCR were given in Liu et al. [27]. PCR products from each sample were purified using an AxyPrep DNA Gel Extraction Kit (Axygen Biosciences, Union City, CA, USA) and quantified using QuantiFluorTM-ST (Promega, USA). Purified amplicons from different samples were pooled in equimolar ratio and subjected to paired-end sequencing (2 × 300) on an Illumina MiSeq platform (Illumina, San Diego, CA, USA) in Majorbio Bio-Pharm Technology Co. Ltd. (Shanghai, China). The procedure of the sequence processing was the same as Liu et al. [27]. Briefly, the raw reads were demultiplexed, quality filtered, and assembled, followed by the identification and removal of chimeric sequences. Operational taxonomic units (OTUs) were obtained using UPARSE (v. 7.1) at a 97% similarity cutoff, and their taxonomy was assigned by RDP Classifier against the SILVA 16S rRNA database (SSU138) with a confidence threshold of 70%. The final OTU table contained 6199–70461 sequences (with a mean of 37603) for different samples (Additional file 2: Table S8).

### Metagenomic sequencing and genome reconstruction

Total genomic DNA was extracted from 10–20 g of sediments from each depth fraction, using the FastDNA® SPIN Kit for Soil (MP Biomedicals, USA). DNA fragment libraries were prepared by shearing genomic DNA from each sample and were then subjected to metagenomic sequencing in the BGI group (Shenzhen, China) using the BGIseq 500 platform, which generated 238.5 Gb of raw reads for the nine metagenomes (23.1–36.8 Gb for individual metagenome, with an average value of 26.5 Gb).

Metagenomic reads from each sample were quality filtered using Trimmomatic v. 0.38 [66] with parameters specified as “LEADING:30 TRAILING:30 CROP:90 HEADCROP:10 SLIDINGWINDOW:4:25 MINLEN:50,” and were separately assembled with IDBA_UD v. 1.1.3 (*k*-mer range 50–80, step 15) [67]. The quality-filtered reads were mapped back to the assemblies using Bowtie2 (v. 2.3.4.1) [68], and coverage was determined according to the mapping results with the jgi_summarize_bam_contig_depths script [68]. Metagenome binning was conducted for assemblies longer than 2500 bp using MetaBAT v. 2.12.1 [69] and CONCOCT v. 1.0.0 [70] with default parameters, and were subsequently refined using Binning_refiner v. 1.2 [71]. Quality of the MAGs was assessed by CheckM v. 1.1.2 using the lineage_wf workflow [72], and only MAGs with completeness > 50% and contamination < 5% were kept for further analysis. Redundant bins were subsequently dereplicated using dRep v. 2.3.2 [73] at 99% average nucleotide identity (ANI) (all other parameters were set to the default), and MAG with the highest quality was selected from each cluster for downstream analysis. The genome size was estimated by dividing the size of the MAG by its estimated completeness.

### Phylogenomic analysis and taxonomic classification

A phylogenomic tree was constructed for the MAGs and 1872 *Chloroflexi* genomes retrieved from the NCBI, JGI and National Genomics Data Center (NGDC) databases (downloaded in April, 2020), using the 43 universal single-copy genes (SCGs) used by CheckM [72]. Protein sequences of the SCGs were identified using HMMER v. 3.1b2 [74] with default parameters, individually aligned with MAFFT v. 7.467 [75] and then concatenated. Phylogenomic tree was constructed based on the alignment using FastTree2 v. 2.1.11 [76], with a JTT model, a gamma approximation and 100 bootstrap replicates.

The closest genomes of each MAG were determined based on their placements in the phylogenomic tree. A final maximum-likelihood phylogenomic tree was then constructed using the MAGs recovered in this study, their closest relatives, all *Chloroflexi* genomes previously reported from trenches, and representative genomes of all known classes of *Chloroflexi*, with *Mycobacterium tuberculosis* (GCA_000195955.2) as outgroup. The phylogenomic tree was visualized using iTOL [77]. Detailed taxonomic classification of the MAGs was determined using GTDB-Tk, which is based on the phylogenetically calibrated Genome Taxonomy Database (GTDB) [29]. The novelty of the MAGs was determined in GTDB-tk based on a combination of their placement in the GTDB reference tree, their RED values, and their average nucleotide identity (ANI) to reference genomes [29, 78]. Briefly, classifications were primarily determined by the placement of the genomes in the GTDB reference tree; if the placement of a genome is ambiguous, the RED value is used for further classification based on well-established standards for each taxonomy level in GTDB; and the assignment of a genome to an existing species was based on the ANI value to reference genomes with a threshold of 95% [29, 78, 79].

### Relative abundance estimation and global distribution

To estimate relative abundance and distribution of recovered MAGs in the sampled hadal sediments, 16S rRNA gene of the MAGs was predicted using barrnap v. 0.9 with default parameters (https://github.com/tseemann/barrnap) and searched with BLASTN [80] against the 16S rRNA and 16S rRNA gene libraries constructed in this study. Only the BLASTN hits with identity > 97%, alignment length > 300 bp, and *e*-value < 1e^−5^ were considered, and the closest matched OTUs were selected. If multiple OTUs were identified as the closest matches for a MAG, the most abundant OTU was selected. The closest-match OTUs were then extracted and their relative abundance in the 16S rRNA and 16S rRNA gene libraries from different layers of the sediment were examined.

The distribution of MAGs in hadal sediments was also estimated via reads recruitment as described by Mehrshad et al. [8]. Briefly, rRNA gene sequences in MAGs were first masked to avoid bias in recruitment results. Recruitments were performed using BLASTN, and hits were filtered with a length cut-off of 50 bp, an identity cut-off of 95%, and an *e*-value cut-off of 1e^−5^ [8]. Qualified hits were used to compute the RPKG (reads recruited per kilobase of genome per gigabase of metagenome) values, which reflect a normalized abundance allowing the comparison across different genomes and metagenomes. Reads recruitment was also applied against 58 publicly available metagenomes derived from microbial communities in seawater and surface sediments from epi-, meso-, bathyl-, and hadal zones of the open ocean, and those from sediments of mud volcano, deep-sea oil spilling sites, deep subseafloor, coastal regions, river, and salt-lake, as well as two metagenomes from soil (Additional file 2: Table S5), to estimate the global distribution of recovered MAGs.

### Gene annotation and metabolic reconstruction

Coding sequences in the MAGs were predicted using Prodigal v. 2.6.3 with default setting [81]. Functional annotation was performed by using BlastKOALA against the KEGG database with default parameters [82], by running similarity searches with BLASTP against the Cluster of Orthologous Groups (COG, December 2014 release) with an *e*-value cut-off of 0.001 [83], as well as by running PROKKA under the “-- metagenome” mode and with kingdom specified as “Bacteria” [84]. Carbohydrate active enzymes were identified using dbCAN 1.0 (default setting) against the CAZy database (version 07312019) [85]. Missing enzymes for pathways of interest were further searched by running tBLASTn against relevant reference sequences in the NCBI database. A tBLASTn hit with an *e*-value ≤ e^−5^, sequence identity ≥ 30%, and a percent alignment length ≥ 30% was considered a potential homolog [86]. The tBLASTn hits were also confirmed by the BLASTp search against the RefSeq protein database [86].

## Supplementary Information

**Additional file 1.** Supplementary figures.

**Additional file 2.** Supplementary tables.

**Additional file 3.** Taxonomy names proposed for the six MAGs qualified as type material.

## Data Availability

The sequences of 16S rRNA and 16S rRNA gene libraries, the metagenomic raw reads of the Mariana Trench sediments, as well as all metagenome-assembled *Chloroflexi* genomes of this study are available in the NCBI database and can be accessed under project ID PRJNA692099.
