# Supplementary material for: Novel Chloroflexi genomes from the deepest ocean reveal metabolic strategies for the adaptation to deep-sea habitats
Source: Microbiome. 2022 May 10;10:75. doi: 10.1186/s40168-022-01263-6 (PMC9088039; doi:10.1186/s40168-022-01263-6)
Supplement: Supplementary file 2 — Additional file 1. Supplementary figures. [file 40168_2022_1263_MOESM2_ESM.docx]

**Additional file 1**

**Novel *Chloroflexi* genomes from the deepest ocean reveal effective metabolic strategies to adapt deep**-**sea habitats**

Rulong Liu^1, 2*^, Xing Wei^1, 2^, Weizhi Song^3^, Li Wang^1, 2^, Junwei Cao^1, 2^, Jiaxin Wu^1, 2^, Torsten Thomas^3^, Tao Jin^4^, Zixuan Wang^5^, Wenxia Wei^1, 2^, Yuli Wei^1, 2^, Haofeng Zhai^1, 2^, Cheng Yao^1, 2^, Ziyi Shen^1, 2^, Jiangtao Du^1, 2^, Jiasong Fang^1, 6, 7*^

^1^ Shanghai Engineering Research Center of Hadal Science and Technology, College of Marine Sciences, Shanghai Ocean University, Shanghai, China

^2^ National Engineering Research Center for Oceanic Fisheries, Shanghai Ocean University, Shanghai, China

^3^ Centre for Marine Science & Innovation and School of Biological Earth and Environmental Science, University of New South Wales, Kensington, Australia

^4^ BGI-Shenzhen, Shenzhen, Guangdong, China.

^5^ Laboratory for Marine Biology and Biotechnology, Qingdao National Laboratory for Marine Science and Technology, Qingdao, China

^6^ Department of Natural Sciences, Hawaii Pacific University, Honolulu, HI, USA

***Correspondence:**

**Rulong Liu**, [rlliu@shou.edu.cn](mailto:rlliu@shou.edu.cn), and **Jiasong Fang**, jsfang@shou.edu.cn

**Supplementary Figures**


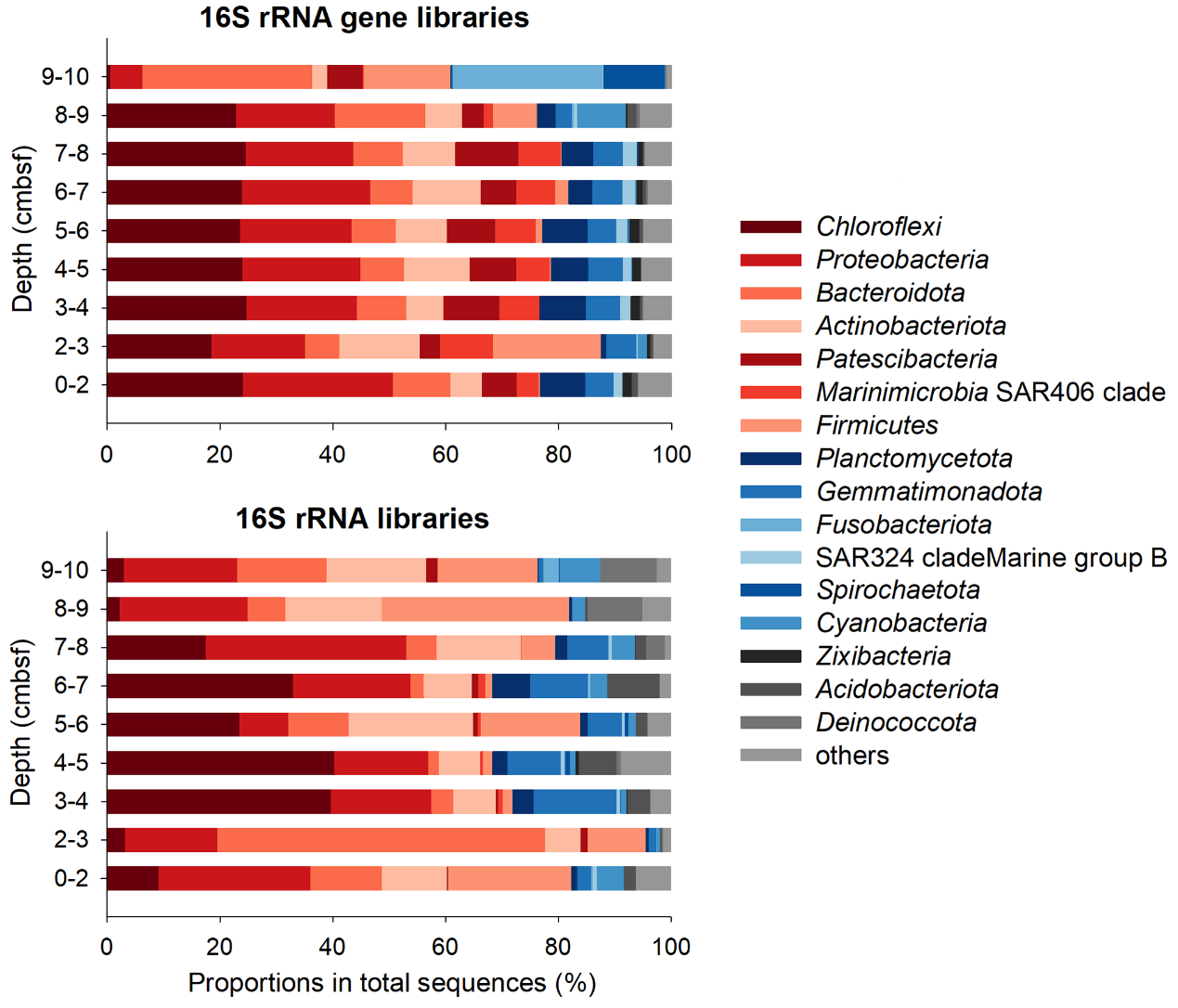


**Figure S1.** Composition of the bulk and potentially active bacterial communities in the hadal sediments at the phylum level, revealed by 16S rRNA gene and 16S rRNA, respectively.


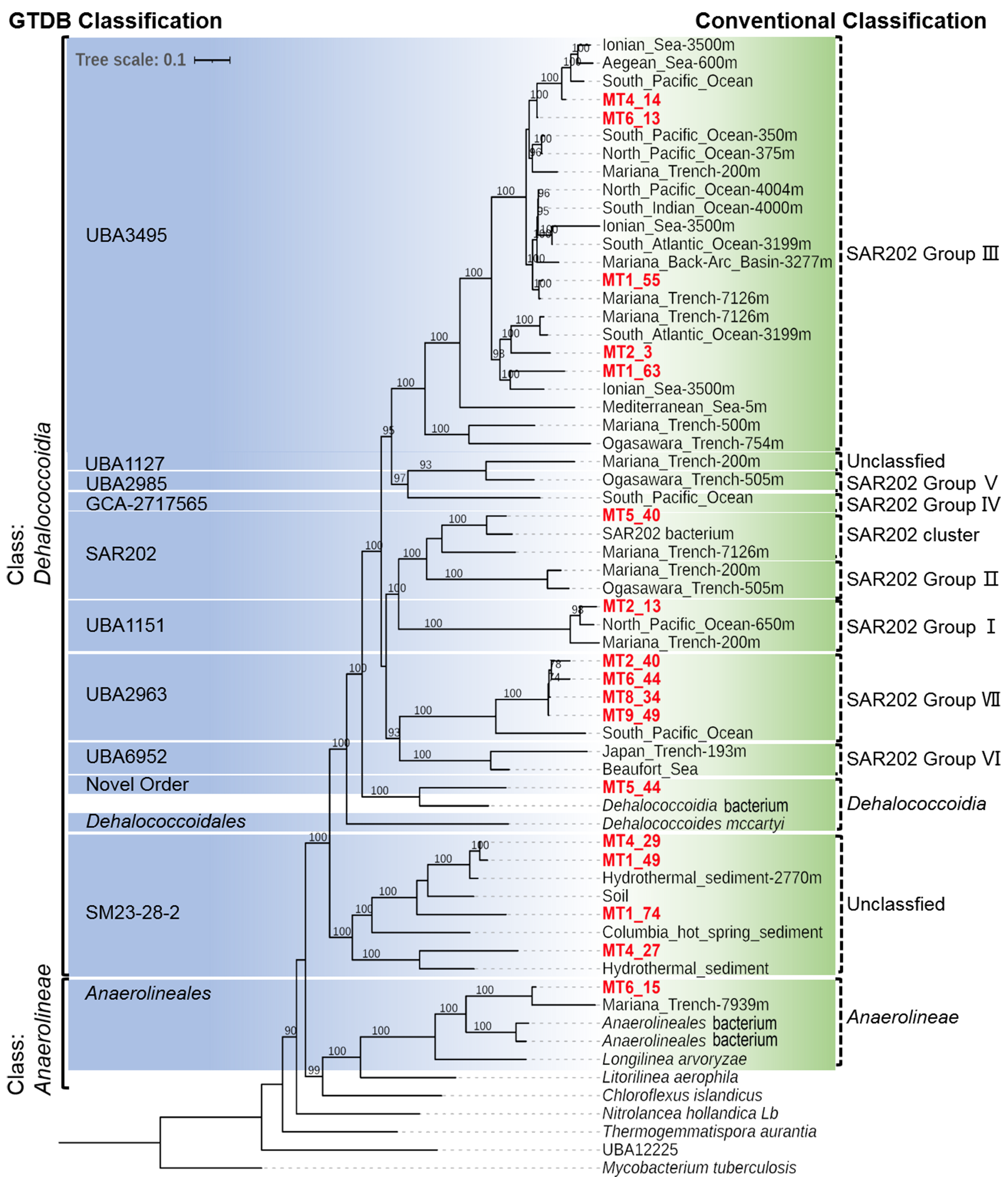


**Figure S2.** **Maximum likelihood phylogenomic tree of the reconstructed *Chloroflexi* MAGs (red color).** The blue background on the left hand side indicates taxonomic classification based on GTDB-tk, and the green background on the right hand side indicates conventional classification (subgroups). Bootstrap values based on 100 replications are shown as percentages on the branches.


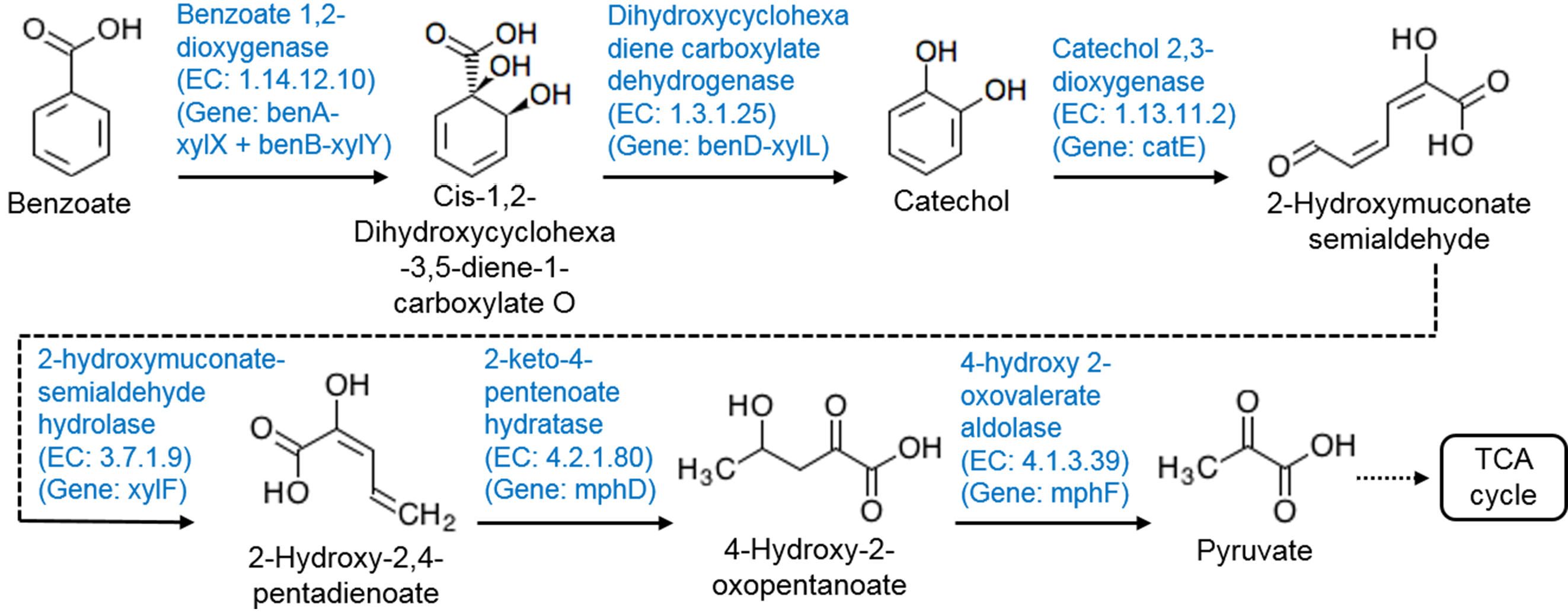


**Figure S3.** **The benzoate degradation pathway annotated in the *Chloroflexi* MAGs.** Blue text indicates the enzymes and their coding genes successfully annotated in the MAGs.


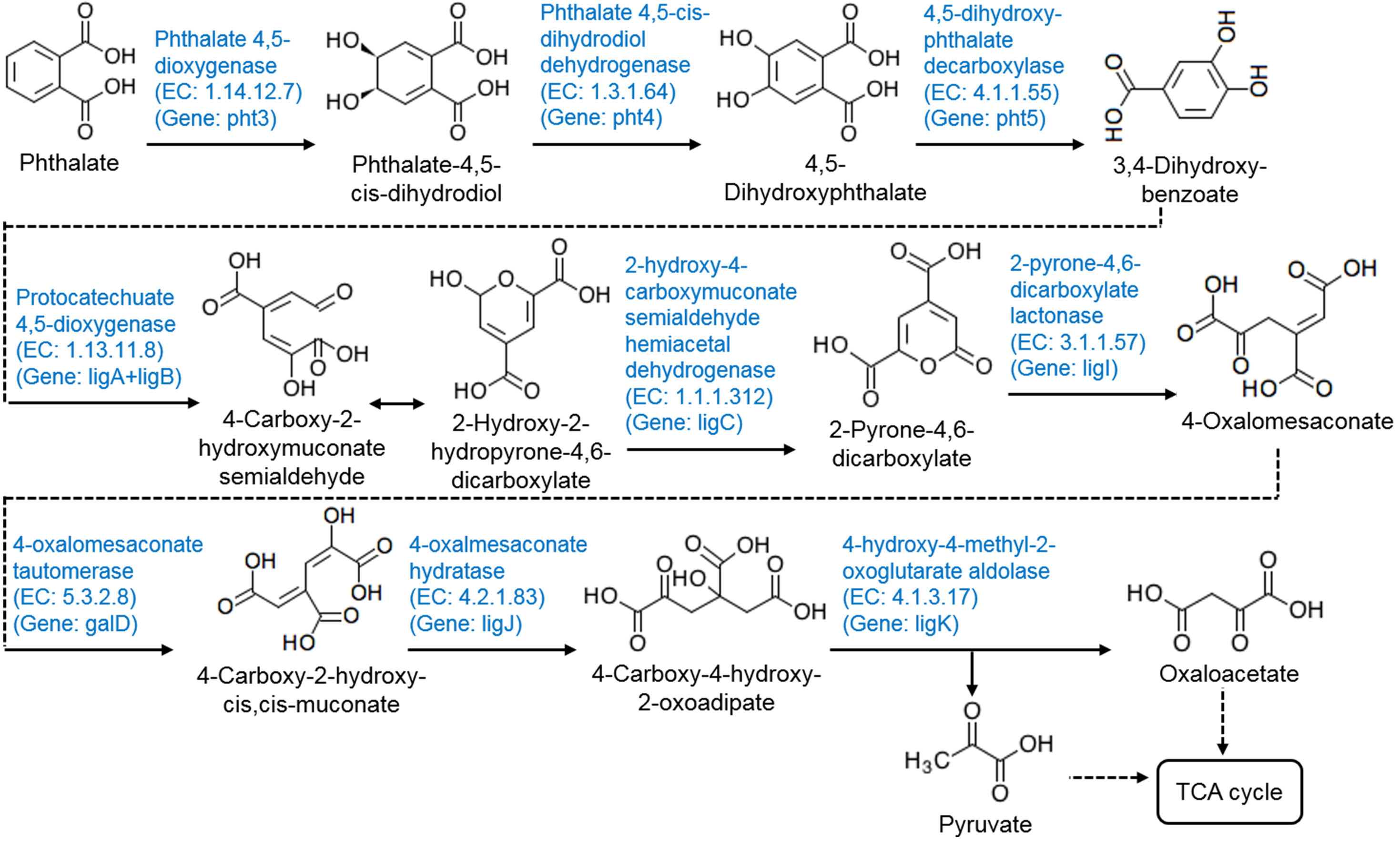


**Figure S4.** **Phthalate degradation pathway annotated in the *Chloroflexi* MAGs.** Blue text indicates the enzymes and their coding genes successfully annotated in the MAGs.


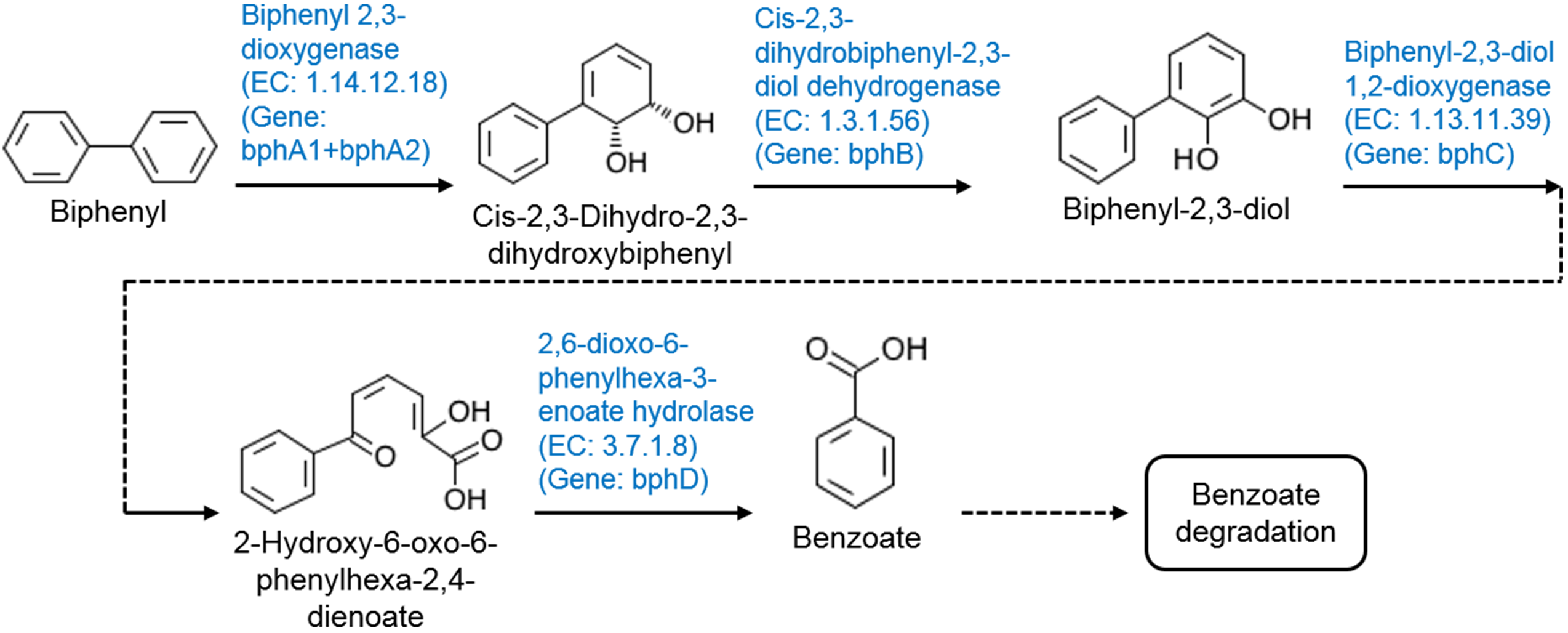


**Figure S5. Biphenyl degradation pathway annotated in the recovered *Chloroflexi* MAGs.** Blue text indicates the enzymes and their coding genes successfully annotated in the MAGs.


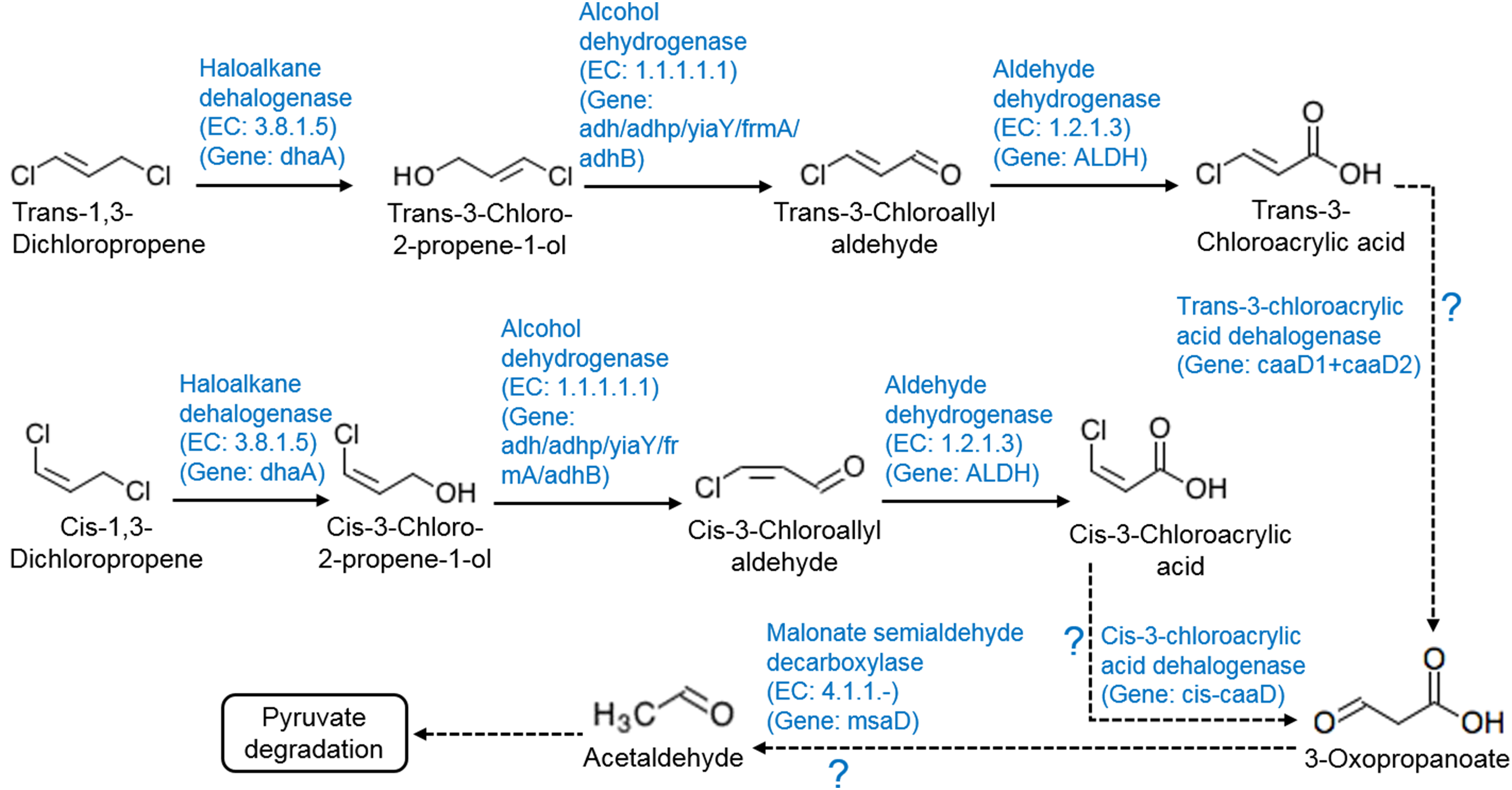


**Figure S6. Chloroalkenes degradation pathways annotated in the recovered *Chloroflexi* MAGs.** Blue text indicates the enzymes and their coding genes successfully annotated in the MAGs, and the missing steps (the last step) were indicated by black dash lines and a question mark.


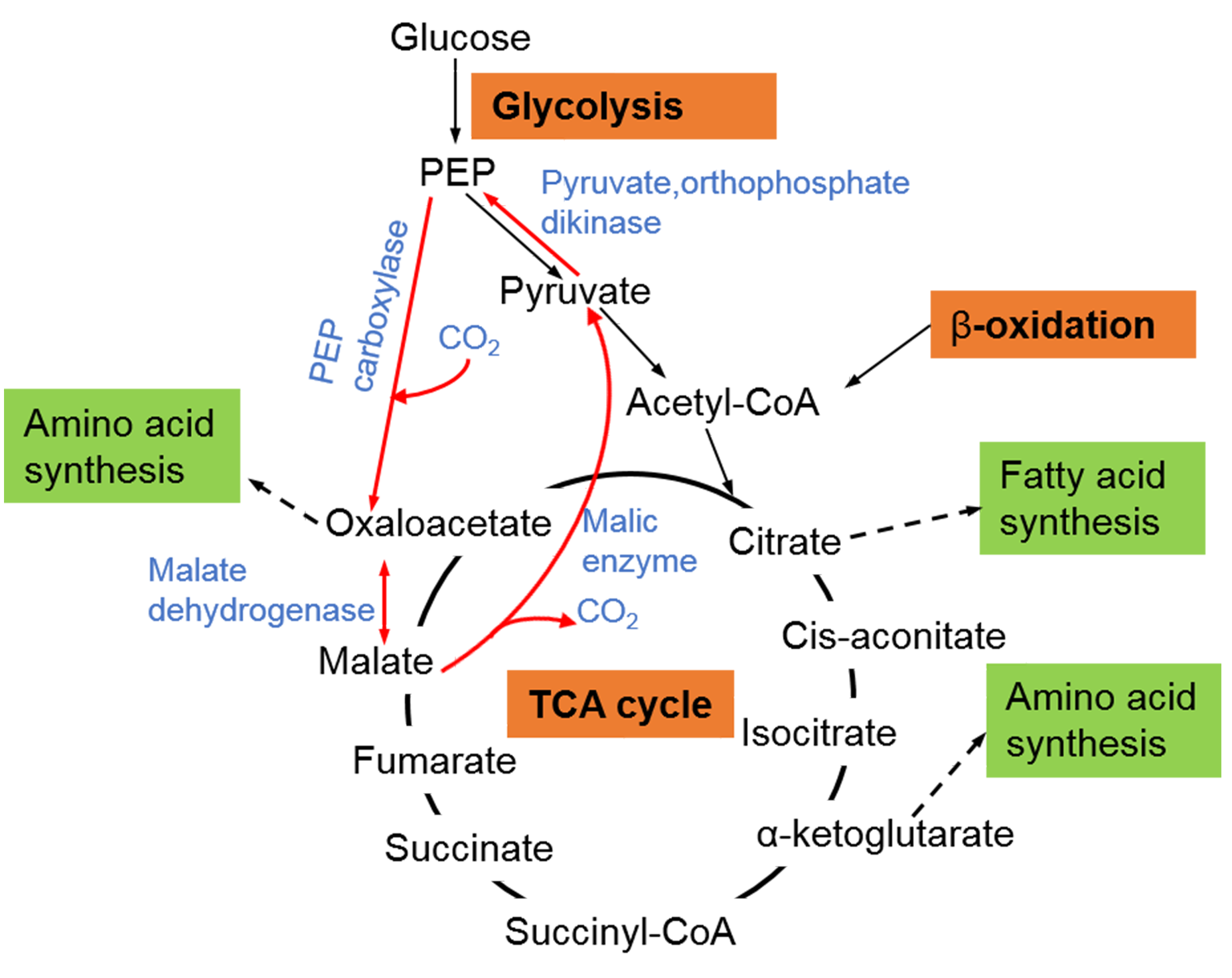


**Figure S7. The phosphoenolpyruvate (PEP)–pyruvate–oxaloacetate node in the recovered *Chloroflexi* MAGs.** Blue text indicates the related enzymes with their genes annotated in the MAGs, while red lines indicate the reactions they catabolized. The links between PEP–pyruvate–oxaloacetate node and central metabolism (black solid lines and orange background) and biosynthesis (black dashed lines and green background) were shown.
