# Supplementary material for: Novel Chloroflexi genomes from the deepest ocean reveal metabolic strategies for the adaptation to deep-sea habitats
Source: Microbiome. 2022 May 10;10:75. doi: 10.1186/s40168-022-01263-6 (PMC9088039; doi:10.1186/s40168-022-01263-6)
Supplement: Supplementary file 4 — Additional file 3. Taxonomy names proposed for the six MAGs qualified as type material. [file 40168_2022_1263_MOESM4_ESM.docx]

**Additional file 3**

**Novel *Chloroflexi* genomes from the deepest ocean reveal metabolic strategies for the adaptation to deep**-**sea habitats**

Rulong Liu^1, 2*^, Xing Wei^1, 2^, Weizhi Song^3^, Li Wang^1, 2^, Junwei Cao^1, 2^, Jiaxin Wu^1, 2^, Torsten Thomas^3^, Tao Jin^4^, Zixuan Wang^5^, Wenxia Wei^1, 2^, Yuli Wei^1, 2^, Haofeng Zhai^1, 2^, Cheng Yao^1, 2^, Ziyi Shen^1, 2^, Jiangtao Du^1, 2^, Jiasong Fang^1, 6, 7*^

^1^ Shanghai Engineering Research Center of Hadal Science and Technology, College of Marine Sciences, Shanghai Ocean University, Shanghai, China

^2^ National Engineering Research Center for Oceanic Fisheries, Shanghai Ocean University, Shanghai, China

^3^ Centre for Marine Science & Innovation and School of Biological Earth and Environmental Science, University of New South Wales, Kensington, Australia

^4^ BGI-Shenzhen, Shenzhen, Guangdong, China.

^5^ Tidal Flat Research Center of Jiangsu Province, Nanjing, Jiangsu, China

^6^ Laboratory for Marine Biology and Biotechnology, Qingdao National Laboratory for Marine Science and Technology, Qingdao, China

^7^ Department of Natural Sciences, Hawaii Pacific University, Honolulu, HI, USA

***Correspondence:**

**Rulong Liu**, [rlliu@shou.edu.cn](mailto:rlliu@shou.edu.cn), and **Jiasong Fang**, jsfang@shou.edu.cn

**Taxonomy names proposed for the six MAGs qualified as type material:**

Six MAGs including MT 6_15, MT 4_27, MT2_13, MT2_3, MT6_13 and MT4_14, showed completeness of >80% and contaminations lower than 3.6% (Table 1), and are qualified as type materials according to the criteria defined recently for taxonomy of uncultivated prokaryotes [1, 2]. Following guidelines developed by Genomics Standards Consortium [3], Konstantinidis et al. [4] and Chuvochina et al. [5], we propose the names *Candidatus* Hastsouellaceae (fam. nov.) and *Ca.* Hastsouella hadalis (genus nov. and species nov.) for MT6_15, for its recovery from the hadal zone and reported by the Hadal Science and Technology Research Centre in Shanghai Ocean University (HAST-SOU). MT 4_27 was putatively named as *Ca.* Dehalosedimentum haloalkanolyticum (genus nov. and species nov.) for its capability of degradation of haloalkanes, and its preferential distribution in deep sea sediments. The higher ranks were named as family “*Ca.* Dehalosedimentaceae” (GTDB family RBG-16-64-32) and order “*Ca.* Dehalosedimentales” (GTDB order SM2-28-2). MT2_13 was putatively named as *Ca.* Dehaloaerobium marianense (species nov.) for its capability to degrade organic halides and recovered from the surface sediment of the Mariana Trench, and the higher ranks were putatively named as genus *Ca.* Dehaloaerobium (GTDB: UBA1328), family *Ca.* Dehaloaerobiaceae (GTDB: Bin127) and order *Ca.* Dehaloaerobiales (GTDB: UBA1151). MT2_3, MT6_13 and MT4_14 belong to the GTDB family UBA3495 of the order UBA3495. Among them, MT6_13 and MT4_14 are representing two novel species within the genus UBA9611, while MT2_3 was representing a novel genus (Table S3). MT2_3 was putatively named as *Ca.* Dehaloaromaticum marinum (genus nov., species nov.) for its capability of degrading halogenated and aromatic compounds. We propose to name the genus UBA9611 *Ca.* Dehalovulgatia for their capability to degrade organic halides and widespread in the deep-sea environments. MT6_13 was named as *Ca.* Dehalovulgatia sulfonica (species nov.) and MT4_14 as *Ca.* Dehalovulgatia fluoreniphila (species nov.) for their capability to degrade sulfonate compounds and fluorene, respectively. The family UBA3495 and order UBA3495 were named *Ca.* Dehalovulgatiaceae *and Ca.* Dehalovulgatiales*,* respectively.

Etymology of the genus names:

Hastsouella L. fem. dim. suff. *-ella*, diminutive ending; N.L. fem. dim. n. *Hastsouella*, arbitrarily formed name from the acronym of the Hadal Science and Technology Research Centre in Shanghai Ocean University (HAST-SOU).

Dehalosedimentum L. prep. *de*, away, off; N.L. pref. *halo-*, halogen; L. neut. n. *sedimentum*, a settling, subsidence; N.L. neut. n. *Dehalosedimentum*, a dehalogenation bacterium preferentially living in deep-sea sediments.

Dehaloaerobium L. prep. *de*, away, off; N.L. pref. *halo-*, halogen; Gr. masc. n. *aêr*, air; Gr. masc. n. *bios*, life; N.L. neut. n. *Dehaloaerobium*, a bacterium performing dehalogenation processes under aerobic conditions.

Dehaloaromaticum L. prep. *de*, away, off; N.L. pref. *halo-*, halogen; L. neut. n. *aromaticum*, aromatic compounds; N.L. neut. n. *Dehaloaromaticum*, a bacterium degrading halogenated and aromatic compounds.

Dehalovulgatia L. prep. *de*, away, off; N.L. pref. *halo-*, halogen; L. masc. part. adj. *vulgatus*, common; N.L. fem. n. *Dehalovulgatia*, a bacterium performing dehalogenation and widespread in the ocean.

**References:**

1. Parks DH, Chuvochina M, Chaumeil PA, Rinke C, Mussig AJ, Hugenholtz P. A complete domain-to-species taxonomy for Bacteria and Archaea. Nat Biotechnol. 2020;38:1079-86.

2. Murray AE, Freudenstein J, Gribaldo S, Hatzenpichler R, Hugenholtz P, Kämpfer P, et al. Roadmap for naming uncultivated Archaea and Bacteria. Nat Microbiol. 2020;5:987-94.

3. Bowers RM, Kyrpides NC, Stepanauskas R, Harmon-smith M, Doud D, Jarett J, et al. Minimum information about a single amplified genome (MISAG) and a metagenome-assembled genome (MIMAG) of bacteria and archaea. Nat Biotechnol. 2017;35:725–31.

4. Konstantinidis KT, Rosselló-Móra R, Amann R. Uncultivated microbes in need of their own taxonomy. ISME J. 2017;11: 2399–406.

5. Chuvochina M, Rinke C, Parks DH, Rappé MS, Tyson GW, Yilmaz P, et al. The importance of designating type material for uncultured taxa. Syst Appl Microbiol. 2019;42:15–21.
